# Cytotoxicity and molecular-docking approach of a new rosane-type diterpenoid from the roots of *Euphorbia nematocypha*

**DOI:** 10.3389/fchem.2022.912738

**Published:** 2022-08-08

**Authors:** Nali Song, Xi Zheng, Jiapeng Wang, Li Zhu, Chengyao Wang, Le Cai, Zhongtao Ding

**Affiliations:** ^1^ Functional Molecules Analysis and Biotransformation Key Laboratory of Universities in Yunnan Province, Key Laboratory of Medicinal Chemistry for Natural Resource, Ministry of Education, School of Chemical Science and Technology, Yunnan University, Kunming, China; ^2^ Central Laboratory, Yunnan Institute of Traditional Chinese Medicine and Materia Medica, Kunming, China; ^3^ State Key Laboratory for Conservation and Utilization of Bio-Resources in Yunnan, Yunnan University, Kunming, China; ^4^ College of Pharmacy, Dali University, Dali, China

**Keywords:** *Euphorbia nematocypha*, diterpenoid, nematocynine, cytotoxicity, molecular docking, DPPH radicals, scavenging

## Abstract

A new rosane-type diterpenoid (**1**) along with nine known diterpenoids (**2–10**), were isolated from the dried roots of *Euphorbia nematocypha*. The absolute configuration was elucidated from spectroscopic (nuclear magnetic resonance, high-resolution electrospray ionization mass spectrometry, and electronic circular dichroism) and optical-rotation analyses. Cytotoxicity and the ability to scavenge 2,2-diphenyl-1-picrylhydrazyl radicals were determined. Compound **1** showed remarkable cytotoxicity against human cancer cell lines (HeLa, CT26, and HCC 1806) *in vitro*. The interaction between compound **1** and proteins of ribosomal S6 kinase was revealed using molecular docking and provided valuable insights into the cytotoxic mechanism of action of compound **1**. The latter could be developed as a pharmaceutical agent in the future.

## 1 Introduction

*Euphorbia* (Euphorbiaceae family) is the largest genus of plants, containing >2000 species worldwide (He et al., 2008). *Euphorbia nematocypha* is one of the earliest recorded species in the “southern Yunnan herb,” which is present in “dalangdu,” a traditional Chinese medicine (TCM) formulation. *E. nematocypha* is distributed widely in Yunnan, Sichuan (province of China), Japan, and Korea (Normile, 2003).

*E. nematocypha* has efficacy in TCM formulations. It can overcome water retention, promote circulation of Qi (Chen and Xu, 2003), remove blood stasis, staunch bleeding, expel sores and carbuncles, dispel pathogenic wind, remove edema, and stop itching (Editorial Committee of Flora Reipublicae Popularis Sinicae, 1997). It can be used as a hemostatic agent for bleeding due to external injury, and as a treatment for tumors, ulcers, edema, abdominal mass, abdominal distension, ascites due to liver cirrhosis, and skin itching (Wu et al., 1992; Chen et al., 2014; Gan et al., 2022). *E. nematocypha* is also used to treat bruises, stiffness, indigestion, toothache, and diabetes mellitus (Roy et al., 2020).

Extracts and various fractions from *E. nematocypha* have shown important biological activities, including anti-respiratory syncytial virus (Huang et al., 2014), activities against Gram-negative and Gram-positive bacteria, and can scavenge 2,2-diphenyl-1-picrylhydrazyl (DPPH) radicals, superoxide radicals, and nitric oxide radicals (Kim et al., 2006). As whitening functional cosmetic, *E. nematocypha* extracts reduce melanin production in B16F10 melanoma cells (Kim et al., 2017).

Diterpenoids are the main constituents in plants of the genus *Euphorbia* (Xu Y. et al., 2021). More than 450 diterpenoids have been isolated from *Euphorbia* species (Xu Y. et al., 2021), which are considered to be taxonomic markers of this genus (Min et al., 2021). Diterpenoids from *E. nematocypha* have shown anticancer activity and antiproliferative effects against cell lines (HL-60, A549, MCF-7, HeLa, and P388), as well as antivirus, anti-inflammatory, and antibacterial activities (Wu et al., 1992; Zhao et al., 1995; He et al., 2008; Yang et al., 2014; Xu Y. et al., 2021).

Here, a new rosane-type diterpenoid named “nematocynine” (**1**) and nine known diterpenoids **(2–10**) were isolated from *E. nematocypha.* The absolute configuration of **1** was identified as (6*R*, 8*R*, 9*S*, and 13*S*)*-* **1** based on one-dimensional (1D) and 2D nuclear magnetic resonance (NMR) spectroscopy and electronic circular dichroism (ECD). Their cytotoxic activity and ability to scavenge DPPH radicals were tested. Molecular-docking studies on protein HCC1806 showed that compound **1** had a binding affinity with ribosomal S6 kinase (RSK), and formed three hydrogen-bonding sites (Asp211, Lys100, and Asp148).

## 2 Materials and methods

### 2.1 General experimental procedures

The chemical reagents we used were of analytical grade and purchased from Xilong Scientific (Guangdong, China). Distilled water was obtained using a Classic UF system (Elga LabWater, High Wycombe, United Kingdom). Acetonitrile and methanol were high-pressure liquid chromatography (HPLC)-grade and obtained from Fisher Scientific (Waltham, MA, United States). Optical rotation was measured on an Autopol Ⅵ system (Rudolph Research Analytical, Hackettstown, NJ, United States). A spectrometer (Nicoletis 10 Magna-IR 550; Thermo Fisher Scientific, Madison, WI, United States) was used for infrared (IR) absorption spectroscopy using KBr pellets. NMR spectra were acquired using DRX 600 (600 MHz for ^1^H and 150 MHz for ^13^C) or DRX 400 (400 MHz for ^1^H and 100 MHz for ^13^C) spectrometers (Bruker, Billerica, MA, United States) employing deuterated solvents (chloroform, methanol, or acetone). A G3250AA system (Agilent Technologies, Santa Clara, CA, United States) was employed for high-resolution electrospray ionization mass spectrometry. GF 254 plates (Qingdao Marine Chemicals, Qingdao, China) were employed and compounds were monitored by thin-layer chromatography (TLC). Silica gels (200–300 mesh and 300–400 mesh; Qingdao Haiyang Chemicals, Qingdao, China) and RP-C_18_ chromatographic packing (Φ40-63 µm; Merck, Whitehouse Station, NJ, United States) were used for chromatography. A Sephadex LH-20 column (GE Healthcare, Piscataway, NJ, United States) was used for column chromatography, and spots were processed with a chromogenic agent (10% H_2_SO_4_ in ethanol) followed by heating and visualization under ultraviolet light. Rotavapor (BÜCHI Labortechnik AG, Flawil, Switzerland) was used to recover and evaporate solvents. Glass columns (120 cm × 22 cm, 90 cm × 8 cm, 70 cm × 8 cm, 185 cm × 3.0 cm, or 168 cm × 2.0 cm) were used for isolation of compounds.

Human cancer cell lines (HCC 1806, ST486, CT26, HeLa, and A549) were digested with trypsin EDTA Solution A (Biological Industries Israel Beit-Haemek, Beit-Haemek, Israel), centrifuged (1200 × *g*, 5 min, room temperature) using Eppendorf tubes (Hamburg, Germany), and cultured in RPMI Medium 1640 (1 × ) (Thermo Fisher) supplemented 10% fetal bovine serum (Biological Industries Israel Beit-Haemek), penicillin (100 U/mL), and streptomycin (100 g/ml). Cells were cultured at 37°C in an atmosphere of 5% CO_2_ and 95% air in a CO_2_ incubator (WCI-180; Wiggens, Straubenhardt, Germany).

Compounds were dissolved in dimethyl sulfoxide (MilliporeSigma, Burlington, MA, United States) before dosing. 3-(4,5-Dimethylthiazol-2-yl)-2,5-diphenyltetrazolium bromide (MTT; Shanghai Macklin Biochemicals, Shanghai, China) was dissolved in phosphate-buffered saline (Biological Industries Beit Haemek), and added to wells after 48 h and mixed evenly with a titer plate shaker (Thermo Fisher). A microplate reader (Epoch 2; Bio-Tek, Winooski, VT, United States) was applied to read absorbance data. DPPH was purchased from Shanghai Macklin Biochemicals. Vitamin C was sourced from Yishengtang (Shanxi, China).

### 2.2 Plant material

The dried roots of *E. nematocypha* were purchased in the Luosiwan pharmacy market (Kunming, China) in July 2017. The medicinal plant was identified by Professor Yang Chen (Guizhou Medical University, Guiyang, China). A voucher specimen (kep-09–13) was deposited in the herbarium of Yunnan University (Kunming, China).

### 2.3 Extraction and isolation of components

The dried roots of *E. nematocypha* (29.00 kg) were powdered and extracted with 95% ethanol (EtOH) under reflux with 35 L of solvent for 4 h (first extraction), 25 L of solvent for 3 h (second extraction), and 20 L of solvent for 3 h (third extraction). After solvent removal under reduced pressure by a rotavapor, a dark residue (1800.00 g) was obtained. The residue was dispersed into warm reverse-osmosis water (10 L), enriched with ethyl acetate (EtOAc, 15 L) four times, and butyl alcohol (*n*-BuOH, 15 L) four times successively. The EtOAc extract (845.97 g) and *n*-BuOH extract (560.40 g) were obtained. The EtOAc extract was submitted to a chromatographic column (120 cm × 22 cm) filled with silica gel (11.6 kg) using a gradient of dichloromethane/methanol (CH_2_Cl_2_/MeOH, 50:1, 20:1, 10:1, 5:1, and 2:1 *v/v*; 120-L each) eluted in turn to obtain five fractions (F1, F2, F3, F4, and F5). The weight of F1, F2, F3, F4, and F5 was 523.52 g, 172.22 g, 77.05 g, 99.06 g, and 144.19 g, respectively. The *n*-BuOH extract (560.4 g) was presented to a chromatographic column (120 cm × 22 cm) filled with silica gel (9.0 kg) and eluted with CH_2_Cl_2_/MeOH (20:1, 10:1, 5:1, and 2:1 *v/v*; 80-L each) to afford four fractions (E1, E2, E3, and E4). The weight of E1, E2, E3, and E4 was 10.00 g, 25.37 g, 57.00 g, and 78.08 g, respectively (Song et al., 2015).

F2 (172.22 g) was submitted to a column (90 cm × 8 cm) filled with C_18_ reversed-phase gel (805.0 g) and eluted with MeOH/H_2_O (40:60, 70:30, and 100:0 *v/v*; 10-L each) in turn, and three fractions (F2a, F2b, and F2c) were obtained. The weight of F2a, F2b, and F2c was 25.62 g, 87.49 g, and 34.21 g, respectively. F2b was subjected to a column (90 cm × 8 cm) filled with silica gel (900.0 g) and ether/ethyl acetate fractions (8:1 *v/v*; 80-L each), which led to 14 fractions (F2b1–F2b14). Fraction F2b10 (1.03 g) was chromatographed on the Sephadex LH-20 column (185 cm × 3.0 cm) with methanol/dichloromethane (MeOH/CH_2_Cl_2_, 2:1 *v/v*; 600 ml). Separated fractions were gathered by an automatic collector and compounds were identified by TLC. Compounds **1** (11.0 mg), **6** (15.0 mg), and **7** (58.1 mg) were obtained (Min et al., 2021). Fraction F2b2 (3.20 g) afforded compound **2** (7.1 mg), fraction F2b12 (850.3 mg) afforded compound **4** (19.0 mg), and fraction F2b14 (2.20 g) afforded compound **8** (8.1 mg).

Fraction F1 (523.52 g) was submitted to two columns (90 cm × 8 cm) filled with C_18_ reversed-phase gel (900.0 g each) and eluted with MeOH/H_2_O (40:60, 70:30, and 100:0 *v/v*; 10-L each) in turn. Four fractions (F1a, F1b, F1c, and F1d) were obtained. Fraction F1d (14.1 g) was submitted to a silica-gel column and eluted with CH_2_Cl_2_/MeOH (45:1) to gather compound **3** (40.0 mg). Similarly, four fractions (F3a, F3b, F3c, and F3d) were obtained from fraction F3. Fraction F3b (14.0 g) was submitted to a silica-gel column and eluted with petroleum ether/ethyl acetate (PE/EtOAc, 6:1 *v/v*; 15.6 L), and compound **5** (7.5 mg) was obtained (Chen et al., 2014).

Fraction E3 (57.0 g) was submitted to a column (70 cm × 8 cm) filled with C_18_ reversed-phase gel (805.0 g) and eluted with MeOH/H_2_O (40:60, 70:30, and 100:0 *v/v*; 10-L each) in turn. Four fractions (E3a, E3b, E3c, and E3d) were obtained. E3a (14.99 g) was submitted to a column filled with silica gel (225 g) and eluent of CH_2_Cl_2_/MeOH (40:1 *v/v*; 15 L) to afford compound **9** (6.5 mg).

Fraction E4 (78.08 g) was submitted to a column (90 cm × 8 cm) filled with C_18_ reversed-phase gel (805.0 g) and eluted with MeOH/H_2_O (40:60, 70:30, and 100:0 *v/v*; 10-L each) in turn. Four fractions (E4a, E4b, E4c, and E4d) were obtained. Fraction E4b (6.0 g) was submitted to a column filled with silica gel (90.0 g) and eluted with CH_2_Cl_2_/MeOH (8:1 *v/v*; 8 L) to afford compound **10** (15.0 mg).

### 2.4 Characterization of compound 1

Nematocynine (**1**): white amorphous powder; [α]^20^_D_ = + 19.60 (*c* 1.0, MeOH) (Supplementary Figure S1); IR (KBr) *ν*_max_: 3430, 2927, 1635, 1382, 1280, and 1146 cm^−1^ (Supplementary Figure S2); ^1^H NMR and ^13^C NMR data were obtained (Supplementary Table S1, Supplementary Figures S3 and S4). 2D NMR results were obtained (Supplementary Figures S5–S8). ESI-MS (negative): *m/z* 285 [M-H]^−^ (Supplementary Figure S9). HR-ESI-MS [M + Na]^+^ ion peak at *m/z* 309.1828 (C_19_H_26_O_2_Na; calcd. For 309.1825) (Supplementary Figure S10).

### 2.5 Calculation of ECD spectra

For each diastereomer of compound **1** (**a** and **b**)**,** conformational searching was undertaken using the molecular mechanics (MM+) method implemented in CONFLEX 8.0 (Scube Scientific Software Solutions, New Delhi, India). Conformers with a Boltzmann population >1% were subjected to geometry optimization at the B3LYP/6-31G (d and p) level of theory in the gas phase. Frequency analyses of optimized conformers were run at the same level of theory to ensure that imaginary frequencies were absent. Then, the optimized conformers were subjected to time-dependent density functional theory (TDDFT) ECD calculations at the B3LYP/6-31G (d and p) level. The solvent effect of methanol solution was considered using the DFT level using the polarizable continuum model (PCM) For each conformer, 10 excited states were calculated using Gaussian 09 (https://gaussian.com) (Frisch et al., 2010). The calculated ECD spectra were obtained by weighing the Boltzmann distribution rate of each conformer using SpecDis (Bruhn et al., 2013; Pu et al., 2017; Li et al., 2021; Shu et al., 2022).

### 2.6 Cytotoxic activities

MTT assays (Liu et al., 2021) were carried out to measure the cytotoxicity of isolated compounds. The malignant triple-negative breast cancer (TNBC) cell line HCC 1806 (Kunming Cell Bank of Type Culture Collection (KCB) catalog number: 2014032 YJ; Research Resource Identifier (RRID): CVCL_1258), human B lymphocyte cell line (ST486) (American Type Culture Collection, CRL-1647; RRID: CVCL_1712), colon cancer cell line (CT26) (Cell Bank of Rio de Janeiro, 0402; RRID: CVCL_7256), human cervical cancer cell line (HeLa) (CLS Cell Lines, 300,194/p772_HeLa; RRID: CVCL_0030), and human lung cancer cell line (A549) (Research Cell Bank, RCB0098; RRID: CVCL_0023) were used. About 5×10^3^ cells/well were seeded and cultured into 96-well microtiter plates (Cai et al., 2020). Twenty-four hours after seeding, cells were treated with compounds (2.5, 5, 10, 20, 40, and 80 μM), positive control, and blank control for 48 h. Then, 20 μL of dissolved MTT solution (5 mg/ml) was added to each well, followed by incubation of cells for 4 h at 37 °C in an incubator containing 5% CO_2_. Remove the culture medium from each well, and dimethyl sulfoxide (150 μL) was added. To dissolve MTT-formazan homogeneously, the 96-well plate was agitated for several seconds. The absorbance of the plate was recorded based on the initial value compared with that of the blank control at 570 nm (Lia et al., 2015). Cisplatin and paclitaxel were the positive control drugs, and three replicate wells were used for each concentration. Origin (www.originlab.com) was used to calculate the half-maximal inhibitory concentration (IC_50_) of each compound (Ferreira et al., 2019; Fang et al., 2021; Wang J. P. et al., 2021).

### 2.7 Molecular docking-based virtual screening

Molecular docking toward p90 RSK was done (Jain et al., 2018). The structure of compound **1** was drawn using ChemDraw 18.0 (https://perkinelmerinformatics.com), and the 3D structure files were transformed using Chem3D 18.0 (https://perkinelmerinformatics.com) (Zheng et al., 2021). Using the protein data bank (PDB; www.rcsb.org), the protein structure of RSK (PDB code: 4NUS) was selected and prepared. Water molecules were deleted and the polar hydrogen atoms, charge, and magnetic field were added before docking. AutoDock Tools 1.5.6 (https://autodock.scripps.edu) was used for molecular docking and processing of ligands and receptors (Zheng et al., 2021; Gan et al., 2022). Active pockets were built and saved as protein data bank (PDB), partial charge (Q), and atom type (T) (PDBQT) files. Then, each active site was docked with the compound (Wang Y. et al., 2021). According to the results of molecular docking, the conformation with the most stable structure and lowest energy was selected and imported into PyMoL 1.8 (https://pymol.org) together with the protein (Shen et al., 2019). A binding model using the 3D diagram of the compound and RSK was obtained by processing (Aronchik et al., 2014; Ahmed et al., 2017; Cui et al., 2022).

### 2.8 DPPH radical scavenging capacities

We wished to determine the ability of the compounds in *E. nematocypha* to scavenge DPPH radicals. Reaction mixtures were cultured in the dark for 30 min at room temperature according to a method described previously with several modifications (Marinova and Batchvarov, 2011). Sample solutions (12.5, 25, 50, 100, 200, and 400 μM) and solutions of vitamin C (3.125, 6.25, 12.5, 25, 50, and 100 μM) were prepared. The blank control did not contain DPPH. The methanol control underwent identical treatment to that of the sample solutions. A microplate reader was used to measure the absorbance at 517 nm. The capacity to scavenge DPPH radicals was calculated using the following equation.

$$\text{Inhibition}\left(\text{\%}\right)=\frac{\left(A_{sample}-A_{blank}\right)-\left(A_{methanol}-A_{blank}\right)}{\left(A_{methanol}-A_{blank}\right)}\;\times 100\text{\%}$$

A_sample_ is the average absorbance of three sample solutions as well as vitamin C. A_blank_ is the average absorbance of the three blank control wells (200 μL of methanol). A_methanol_ is the average absorbance of three wells containing 100 μL of methanol and 100 μL of DPPH. A_sample_ is the average absorbance of three wells containing 100 μL of sample and 100 μL of DPPH. The final volume of each well was identical.

## 3 Results and discussion

**FIGURE 1:**
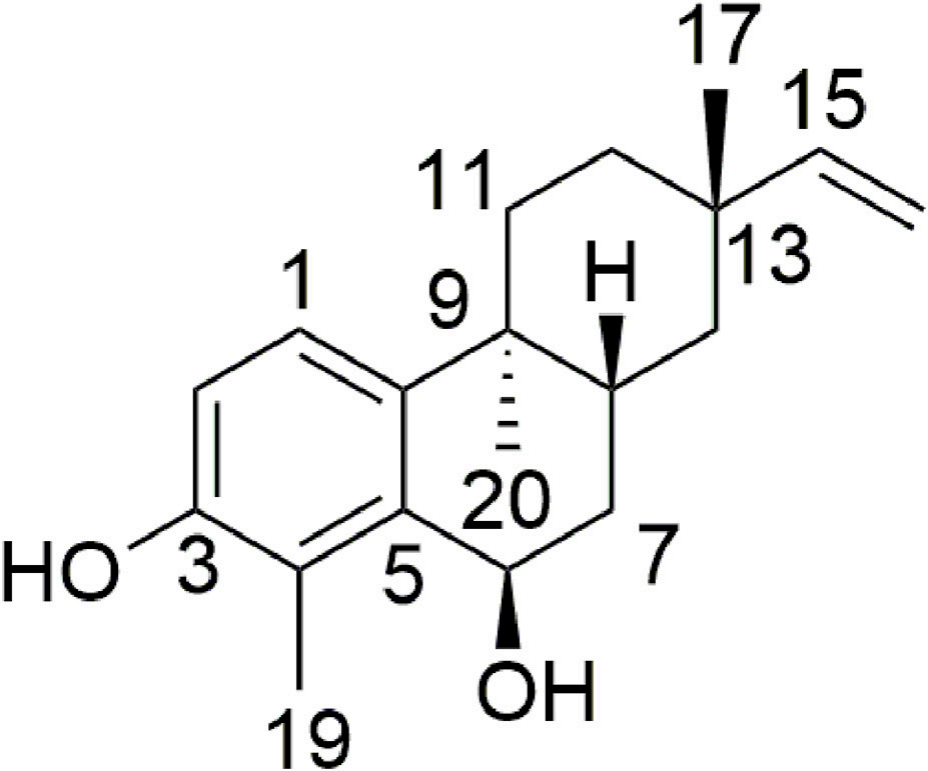
Structure of compound **1** isolated from *E. nematocypha*.

In the separation process, a customized silica-gel column (120 cm × 22 cm) and mounts of reverse-phase silica gel (RP-18; Merck) in a column (90 cm × 8 cm) were used. In addition, a series of diterpenoids were isolated using reverse-phase silica-gel column chromatography with MeOH/H_2_O (40:60 and 70:30 *v/v*). These isolation methods were summarized by our research team recently. As a result, a new rosane-type diterpenoid (**1**) (Figure 1) along with nine known diterpenoids (**2–10**) were isolated and identified.

### 3.1 Identification of compounds

Compound **1** was isolated as a white amorphous powder of molecular formula C_19_H_26_O_2_ based on its positive ion at *m/z* 309.1828 [M + Na]^+^ (calcd. for C_19_H_26_O_2_Na, 309.1825) (Figure 1). IR spectroscopy revealed hydroxyl groups (3430 and 2927 cm^−1^) and double bonds (1635, 1382, 1280, and 1146 cm^−1^). The ^1^H NMR spectrum of compound **1** displayed signals for a benzene ring (*δ*_H_ 7.08, d, *J* = 8.5 Hz and *δ*_H_ 6.75, d, *J* = 8.5 Hz), along with a terminal double bond (*δ*_H_ 5.87, dd, *J* = 17.5, 10.7 Hz, *δ*_H_ 4.97, dd, *J* = 17.5, 1.2 Hz, and *δ*_H_ 4.89, dd, *J* = 10.7, 1.3 Hz). The ^13^C NMR and distortion-less enhancement by polarization transfer (DEPT) spectra of **1** (Supplementary Table S1, Supplementary Figures S3–S8) disclosed 19 carbons: three methyl (*δ*_C_ 23.2, 21.2, and 11.3), five methylene (including one *sp* ^*2*^ carbon) (*δ*_C_ 35.3, 33.9, 33.1, 39.2, and 109.1), five methine (containing three *sp* carbons) (*δ*_C_ 123.3, 115.3, 65.5, 31.1, and 151.1), and six quaternary (*δ*_C_ 152.3, 123.7, 135.7, 36.7, 141.0, and 37.2). This 1D NMR information indicated that compound **1** was an 18-norrosane diterpenoid with an aromatic A-ring. A detailed comparative analysis of NMR spectra between compound **1** and ebraphenol B showed that the NMR signals of C-6 in compound **1** were *δ*_H_ 4.93 (dd, 6.0 Hz, 1.5 Hz) and *δ*_C_ 65.5 (d), whereas the NMR signals of C-6 in ebraphenol B were *δ*_H_ 4.27 (dd, 3.6 Hz, 1.2 Hz) and *δ*_C_ 74.3 (d) (Liu et al., 2014; Lu et al., 2019). In addition, –OCH_3_ signals in compound **1** were absent. These differences implied that C-6 in compound **1** was substituted by –OH.

**FIGURE 2:**
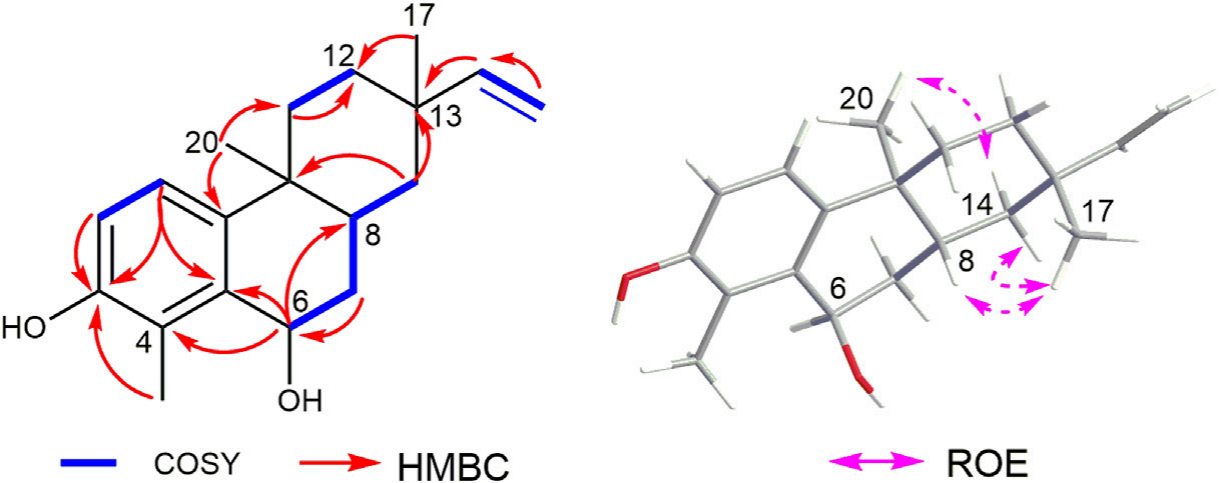
Key ^1^H–^1^H COSY, HMBC, and ROESY correlations of compound **1**.

The heteronuclear multiple bond correlation (HMBC) from H-6 (*δ*_H_ 4.93) to C-5 (*δ*_C_ 136.0) and C-4 (*δ*_C_ 123.7) confirmed the linkage between C-5 (*δ*_C_ 136.0) and C-6 (*δ*_C_ 65.5) (Figure 2). The nuclear Overhauser effect (NOE) correlation of H_3_-17 (*δ*_H_ 1.07) with H-8 (*δ*_H_ 2.17) indicated that CH_3_-17 (*δ*_C_ 11.3) and H-8 (*δ*_H_ 2.17) had an identical orientation. The absence of NOE correlation between H_3_-20 (*δ*_H_ 0.96) and H-8 (*δ*_H_ 2.17) indicated that CH_3_-20 (*δ*_C_ 21.2) had the opposite orientation. However, the relative configuration of C-6 (*δ*_C_ 65.5) could not be identified because the key NOE correlation was lost. Thus, there were four possible absolute configurations for **1** (6*R*, 8*R*, 9*S*, and 13*S*)-**1**; (6*S*, 8*R*, 9*S*, and 13*S*)-**1**; (6*R*, 8*S*, 9*R*, and 13*R*)-**1**; (6*S*, 8*S*, 9*R*, and 13*R*)-**1** (Supplementary Figure S11).

**FIGURE 3:**
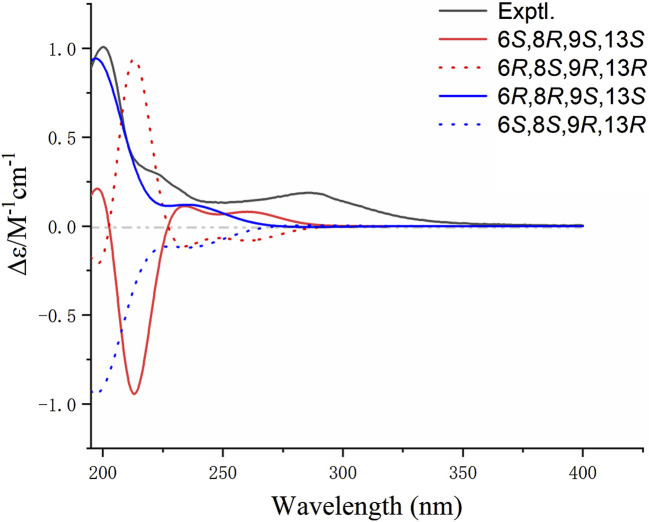
Experimental and calculated ECD spectra of compound **1**.

To further determine the absolute configuration of **1**, the ECD spectra of (6*S*, 8*R*, 9*S*, and 13*S*)-**1** (**1a**) and (6*R*, 8*R*, 9*S*, and 13*S*)-**1** (**1b**) were calculated and compared with the configuration obtained experimentally. According to conformational searches and geometry optimization, three conformers for **1a** and eight conformers for **1b** were obtained. The TDDFT calculation showed that the calculated ECD curve of **1b** matched closely with the experimental spectrum (Figure 3). Thus, the absolute configuration of **1** was identified to be 6*R*, 8*R*, 9*S*, and 13*S*. The key transitions, excitation energies, oscillator, and rotatory strengths contributing to the ECD spectra of the dominant conformers of **1b** were investigated (Supplementary Tables S2–S9). Therefore, the structure of **1** was identified and named nematocynine.

Nine known diterpenoids (**2–10**) were isolated from the dried roots of *E. nematocypha* (Supplementary Figure S12): euphebracteolatin A (**2**) (Mu et al., 2013), Yuexiandajisu D (**3**) (Fu et al., 2006), 11*β*-hydroxy-ent-abieta-8(14),13(15)-dien-16,12*β*-olide (**4**) (Yang et al., 2021), fischeriolide C (**5**) (Lee et al., 2016), fischeriolide B (**6**) (Lee et al., 2016), ebractenoid P (**7**) (Bai et al., 2018), 3*β*,19-dihydroxy-1 (10),15-rosadien-2-one (**8**) (Deng et al., 2010), langduin F (**9**) (Wang et al., 2010; Liang et al., 2014), and ingenol (**10**) (Halaweish et al., 2002). The structures of compounds **2–10** were identified by comparison with the corresponding references listed above, whose NMR spectra were supported (Supplementary Figures S13–S30).

### 3.2 Cytotoxic activity against human cancer cell lines

The cytotoxicity of isolated diterpenoids was evaluated. Compound **1** showed remarkable inhibitory activity against HCC 1806, CT26, and HeLa cells with IC_50_ of 16.96 ± 0.16, 52.04 ± 1.96, and 52.70 ± 0.52 μM, respectively (Table 1). Compound **3** had inhibitory activity against HCC 1806, ST486, and CT26 cells with IC_50_ of 26.46 ± 4.59, 49.31 ± 4.17, and 34.33 ± 12.82 μM, respectively. Compound **6** exhibited pronounced inhibitory activity toward ST486 and A549 cells with IC_50_ of 65.37 ± 22.29 and 75.37 ± 9.89 μM, respectively. Compound **10** displayed inhibitory activity against HCC1806 cells with IC_50_ of 62.49 ± 8.60 μM.

**TABLE 1:** IC_50_ of compounds **1–10** against five tumor cell lines.

Compound	Tumor cell lines (IC_50_, μM)^a^				
	HCC1806	ST486	CT26	HeLa	A549
1	16.96 ± 0.16	60.94 ± 0.74	52.04 ± 1.96	52.70 ± 0.52	>80
2	>80	>80	>80	>80	>80
3	26.46 ± 4.59	49.31 ± 4.17	34.33 ± 12.82	>80	>80
4	>80	>80	>80	>80	>80
5	>80	>80	>80	>80	>80
6	>80	65.37 ± 22.29	>80	>80	75.37 ± 9.89
7	>80	>80	>80	>80	>80
8	>80	>80	>80	>80	>80
9	>80	>80	>80	>80	>80
10	62.49 ± 8.60	>80	>80	>80	>80
Cisplatin	3.77 ± 0.087	1.06 ± 0.029	3.57 ± 0.16	3.90 ± 0.14	9.65 ± 0.55
Paclitaxel	0.042 ± 0.008	4.35 ± 0.37	13.01 ± 1.73	0.33 ± 0.036	7.74 ± 0.93

a IC_50_ data represent three replicates and are shown as the mean ± SD.

### 3.3 Molecular docking on protein HCC1806

RSK has important roles in the survival, growth, translation, and cell cycle of tumor cells (Casalvieri et al., 2017). Abnormal expression of RSK has a close relationship with several tumor types, including TNBC (Zhao et al., 2016; Yoon et al., 2021), colorectal cancer (Xu J. et al., 2021), and lung cancer (Poomakkoth et al., 2016; Casalvieri et al., 2017). To predict the cytotoxicity of compound **1**, molecular docking on RSK protein was undertaken. As one of the most selective and potent RSK inhibitors, LJH685 can inhibit cellular RSK activity (Aronchik et al., 2014). Hence, LJH685 was used to inhibit RSK activity (Jain et al., 2015; Cui et al., 2022). The binding energy (in kcalmol^−1^) of compound **1** for RSK was −8.64, and it was −7.95 for LJH685. Hence, compound **1** possessed lower binding energy and comparative affinity to that of LJH685.

**FIGURE 4:**
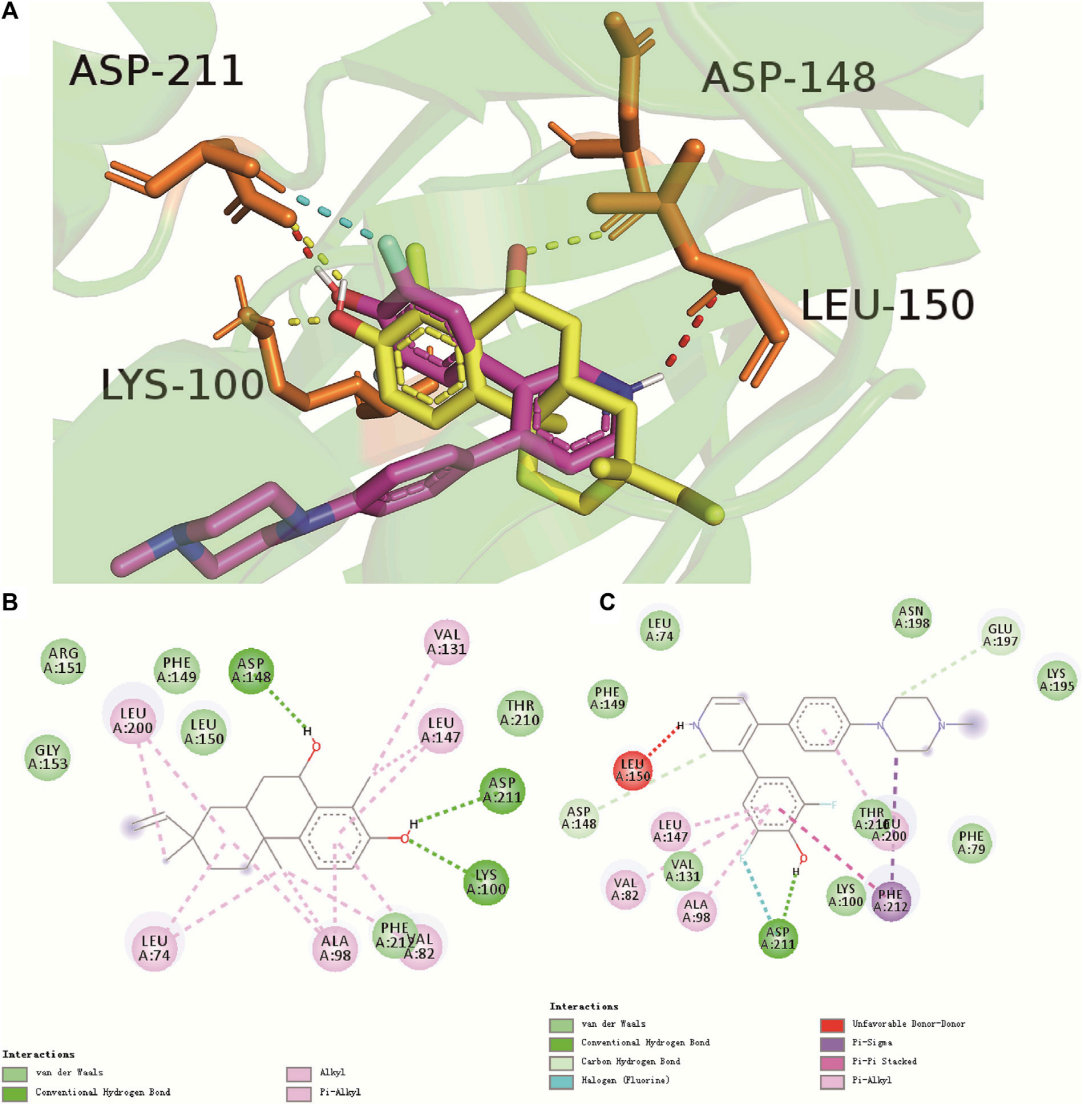
Binding mode of compound **1** (yellow) and LJH685 (purple) against RSK (PDB ID: 4NUS). **(A)** Three-dimensional overlaid pose diagrams. The yellow dotted line signifies the hydrogen bond of compound **1**, red and blue dotted lines represent the hydrogen bond of LJH685. **(B)** Two-dimensional binding modes of compound **1** and RSK. **(C)** Two-dimensional binding modes of LJH685 and RSK. Green lines represent the hydrogen-bonding interaction, aqua blue signifies van der Waals forces, and lilac represents alkyl and Pi-alkyl groups.

The binding site between compound **1** and RSK is illustrated in Figure 4. The hydrogen and oxygen in the hydroxyl group above the benzene ring of compound **1** formed hydrogen bonds with Asp211 (2.3 Å) and Lys100 (2.0 Å), respectively. The hexatomic ring formed a hydrogen bond with the hydroxyl oxygen of Asp148 (2.5 Å). Moreover, hydrophobic interactions and van der Waals forces were responsible for the binding energy with amino-acid residues (Figures 4A,B). The hydrogen on the pyridine nitrogen of LJH685 formed a hydrogen bond with Leu150 (2.8 Å) (Jain et al., 2018; Cui et al., 2022). The hydroxyl group on the benzene ring of hydrogen in LJH685 bonded with Asp211 (1.7 Å). In addition, the fluorine on the benzene ring formed hydrogen bonds with Asp211 (2.1 Å) (Figures 4A,C) but did not form hydrogen bonds with Lys100 as suggested in the literature (Bulusu and Desiraju, 2019; Cui et al., 2022).

Looking at the binding site of compound **1** and LJH685 with RSK, it became obvious that compound **1** had an identical binding mode but additional contacts that contributed to the binding energy and increased potency. Compound **1** had three hydrogen bonds and was well-matched in the RSK pocket.

To study the mechanism of cytotoxicity of compound **1**, the interaction of compound **1** with CT26 cells (PDB code: 4OAS) was carried out (Zhang et al., 2017). The hydrogen on the hexatomic ring formed hydrogen bonds with Leu-54 (1.8 Å). Docking of compound **1** with the proteins from HeLa cells (PDB code: 6VR1) (Batool et al., 2022) showed that the oxygen on the hexatomic ring formed a hydrogen bond with His-188 (1.7 Å) and that bonding was weak.

### 3.4 Ability to scavenge DPPH radicals

The ability of the compounds within *E. nematocypha* to scavenge DPPH radicals is displayed in Table 2. Vitamin C was the positive control drug with IC_50_ of 0.17 ± 0.01 μM. Compounds **1**, **2**, and **4** could scavenge DPPH radicals. Compound **4** showed a good ability to scavenge DPPH radicals, with IC_50_ of 32.38 ± 1.92 μM. Compounds **1** and **2** exhibited moderate antioxidant activity, with IC_50_ of 427.64 ± 8.47 and 57.55 ± 1.59 μM, respectively.

**TABLE 2:** Ability of compounds **1–10** to scavenge DPPH radicals.

Compound	(IC_50_, μM)^a^	Compound	(IC_50_, μM)^a^
1	427.64 ± 8.47	7	>400
2	57.55 ± 1.59	8	>400
3	>400	9	>400
4	32.38 ± 1.92	10	>400
5	>400	Vitamin C	0.17 ± 0.01
6	>400		

a IC_50_ data represent three replicates and are shown as the mean ± SD.

## 4 Conclusion

We described a new rosane-type diterpenoid and nine known diterpenoids isolated from *E. nematocypha*. Through the screening of five human cancer cell lines, compound **1** showed remarkable inhibitory activity against HCC 1806, CT26, and HeLa cells. Compounds **2** and **4** exhibited moderate inhibitory activity *in vitro*. The molecular-docking study of compound **1** with RSK suggested that compound **1** might be an efficacious inhibitor of human breast cancer cells. Compounds **2** and **4** were strong scavengers of DPPH radicals.

## Data Availability

The datasets presented in this study can be found in online repositories. The names of the repository/repositories and accession number(s) can be found in the article/Supplementary Material.
