# Supplementary material for: Cytotoxicity and molecular-docking approach of a new rosane-type diterpenoid from the roots of Euphorbia nematocypha
Source: Front Chem. 2022 Aug 8;10:912738. doi: 10.3389/fchem.2022.912738 (PMC9393309; doi:10.3389/fchem.2022.912738)
Supplement: Supplementary file 1 [file DataSheet1.pdf]

Supplementary Material

Table of Contents

Figure S1. Optical rotation measurement of compound 1. 4

Figure S2. Infrared spectrum (CDCl3) of compound 1 5

Figure S3. ^{1}H NMR spectrum ($CDCl_3$, 400 MHz) of compound 1. 6

Figure S4. ^{13}C and DEPT spectra ($CDCl_3$, 100 MHz) of compound 1................ 7

Figure S5. HSQC spectrum of compound 1. 8

Figure S6. HMBC spectrum of compound 1. 9

Figure S7. ^{1}H-^{1}H COSY spectrum of compound 1. 10

Figure S8. ROESY spectrum of compound 1 11

Figure S9. ESI-MS (negative mode) of compound 1. 12

Figure S10. HR-ESI-MS (positive mode) of compound 1 13

Figure S11. Four possible absolute configurations of compound 1. 14

Figure S12. Structures of the known compounds 2-10. 15

Figure S13. ^{1}H NMR spectrum ($CDCl_3$, 400MHz) of compound 2. 16

Figure S14. ^{13}C and DEPT spectra ($CDCl_3$, 100MHz) of compound 2............. 17

Figure S15. ^{1}H NMR spectrum (CD_3COCD_3, 400MHz) of compound 3. 18

Figure S16. ^{13}C and DEPT spectra (CD_3COCD_3, 100MHz) of compound 3. ... 19

Figure S17. ^{1}H NMR spectrum (CD_3OD, 600MHz) of compound 4................. 20

Figure S18. ^{13}C and DEPT spectra (CD_3OD, 150MHz) of compound 4. 21

Figure S19. ^{1}H NMR spectrum (CD_3OD, 600MHz) of compound 5................. 22

Figure S20. ^{13}C and DEPT spectra (CD_3OD, 150MHz) of compound 5. 23

Figure S21. ^{1}H NMR spectrum ($CDCl_3$, 400MHz) of compound 6 24

Figure S22. ^{13}C and DEPT spectra ($CDCl_3$, 100MHz) of compound 6. 25

Figure S23. ^{1}H NMR spectrum ($CDCl_3$, 400MHz) of compound 7. 26

Figure S24. ^{13}C and DEPT spectra ($CDCl_3$, 100MHz) of compound 7............. 27

Figure S25. ^{1}H NMR spectrum (CDCl3, 600MHz) of compound 8.................. 28

Figure S26. ^{13}C and DEPT spectra ($CDCl_3$, 150MHz) of compound 8............. 29

Figure S27. ^{1}H NMR spectrum ($CDCl_3$, 600MHz) of compound 9. 30

Figure S28. ^{13}C and DEPT spectra ($CDCl_3$, 150MHz) of compound 9............. 31

Figure S29. ^{1}H NMR spectrum (CDCl3, 600MHz) of compound 10................ 32

Figure S30. 13C and DEPT spectra (CDCl3, 150MHz) of compound 10......... 33

Table S1. ^{1}H and ^{13}C NMR Data of Compound 1 and ebraphenol B (δ in ppm, data obtained in $CDCl_3$).. 34

Table S2. Key transitions, oscillator strengths, and rotatory strengths in the ECD spectrum of conformer 1b-1 at the B3LYP/6-31G (d, p) level of theory in MeOH with IEFPCM solvent model... 35

Table S3. Key transitions, oscillator strengths, and rotatory strengths in the ECD spectrum of conformer 1b-2 at the B3LYP/6-31+G(d,p) level of theory in MeOH with IEFPCM solvent model... 37

Table S4. Key transitions, oscillator strengths, and rotatory strengths in the ECD spectrum of conformer 1b-3 at the B3LYP/6-31G (d, p) level of theory in MeOH with IEFPCM solvent model... 41

Table S5. Key transitions, oscillator strengths, and rotatory strengths in the ECD spectrum of conformer 1b-4 at the B3LYP/6-31G (d, p) level of theory in MeOH with IEFPCM solvent model... 44

Table S6. Key transitions, oscillator strengths, and rotatory strengths in the ECD spectrum of conformer 1b-5 at the B3LYP/6-31G (d, p) level of theory in MeOH with IEFPCM solvent model... 47

Table S7. Key transitions, oscillator strengths, and rotatory strengths in the ECD spectrum of conformer 1b-6 at the B3LYP/6-31G (d, p) level of theory in MeOH with IEFPCM solvent model. 51

Table S8. Key transitions, oscillator strengths, and rotatory strengths in the ECD spectrum of conformer 1b-7 at the B3LYP/6-31G (d, p) of theory in MeOH with IEFPCM solvent model. 55

Table S9. Key transitions, oscillator strengths, and rotatory strengths in the ECD spectrum of conformer 1b-8 at the B3LYP/6-31G (d, p) level of theory in MeOH with IEFPCM solvent model. 58

Rudolph Research Analytical

This sample was measured on an Autopol VI, Serial #91058
Manufactured by Rudolph Research Analytical, Hackettstown, NJ, USA.

Measurement Date : Sunday, 02-JAN-2022

Set Temperature : 20.0

Time Delay : Disabled

Delay between Measurement : Disabled

n	Average	Std.Dev.	% RSD	Maximum	Minimum
5	19.60	0.89	4.54	21.00	19.00

S.No	Sample ID	Time	Result	Scale	OR °Arc	WLG.nm	Lg.mm	Conc.g/100ml	Temp.
1	D01	10:59:14 AM	21.00	SR	0.021	589	100.00	0.100	20.3
2	D01	10:59:20 AM	20.00	SR	0.020	589	100.00	0.100	20.2
3	D01	10:59:27 AM	19.00	SR	0.019	589	100.00	0.100	20.1
4	D01	10:59:33 AM	19.00	SR	0.019	589	100.00	0.100	20.0
5	D01	10:59:39 AM	19.00	SR	0.019	589	100.00	0.100	20.0

Figure S1. Optical rotation measurement of compound **1**.

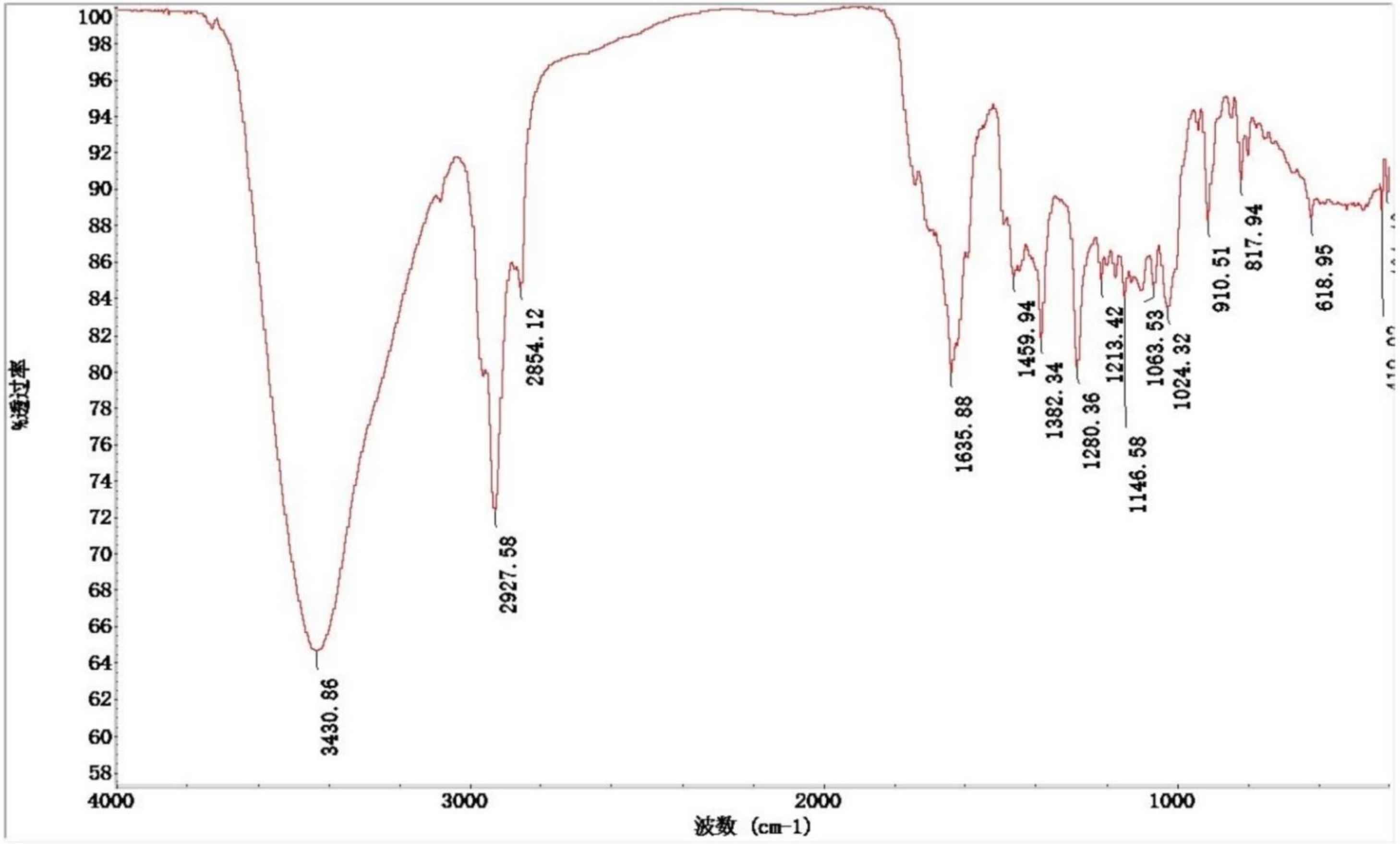

Figure S2. Infrared spectrum (CDCl3) of compound 1.

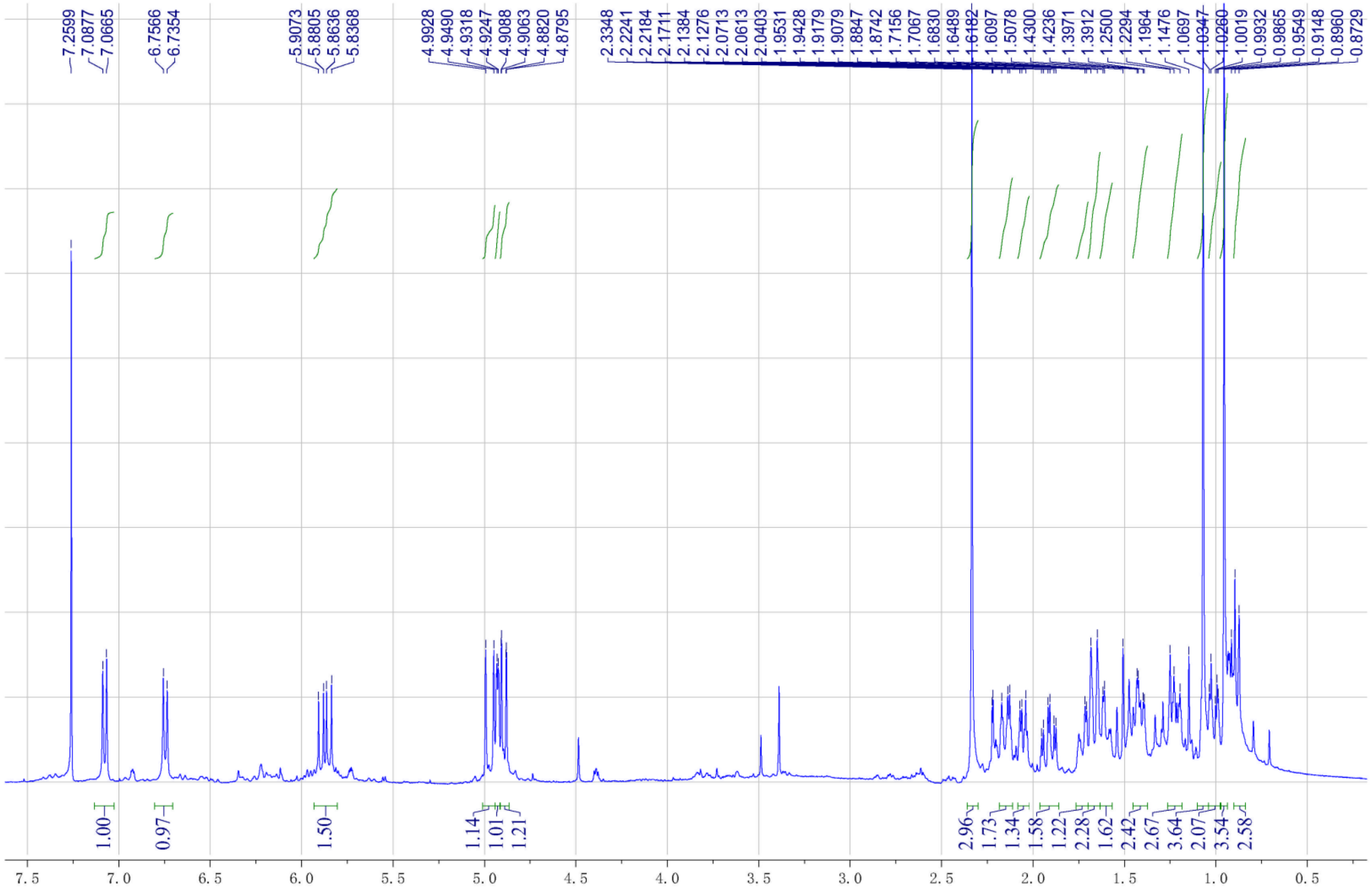

Figure S3. ^{1}H NMR spectrum ($CDCl_3$, 400 MHz) of compound **1**.

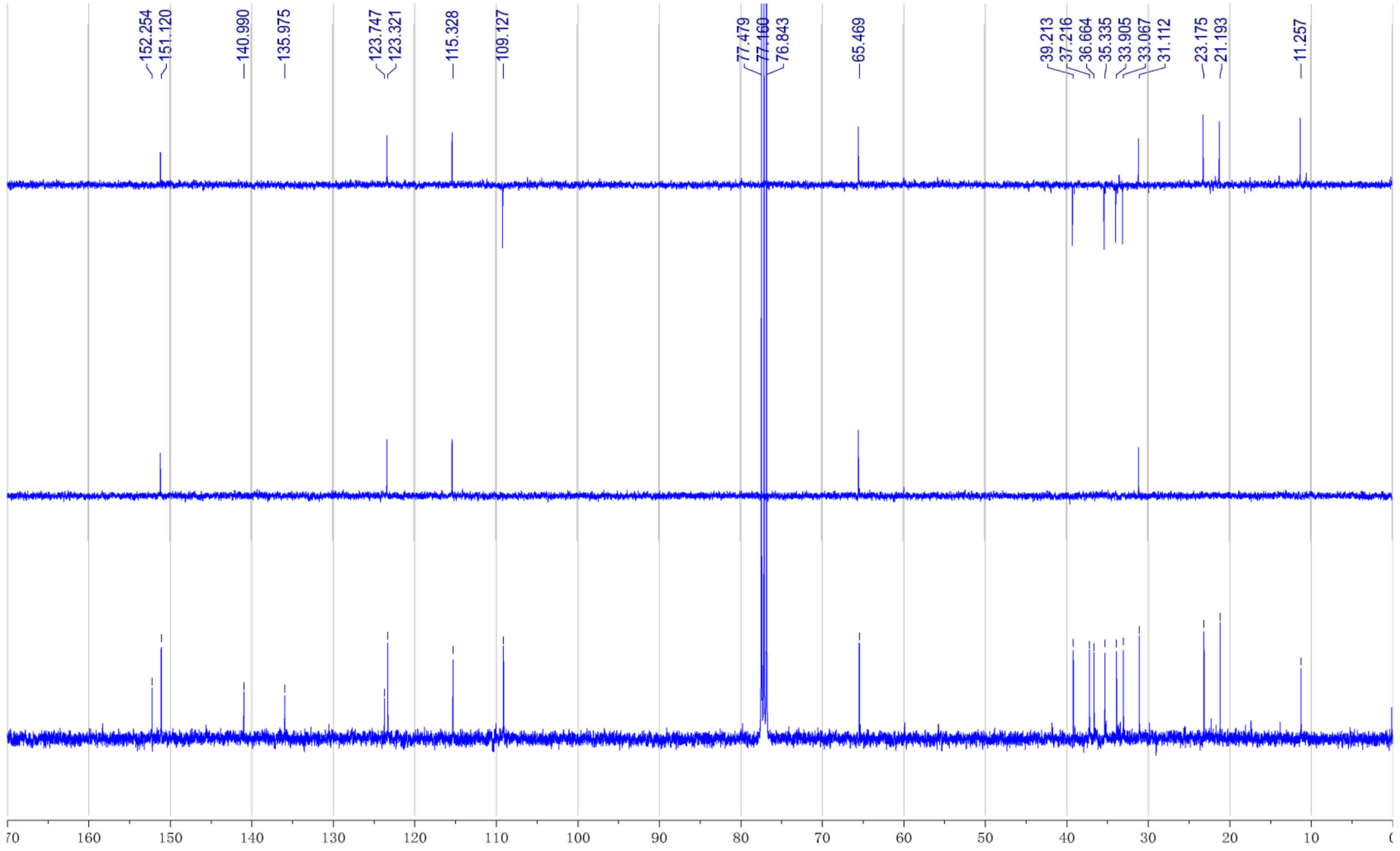

Figure S4. ^{13}C and DEPT spectra ($CDCl_3$, 100 MHz) of compound **1**.

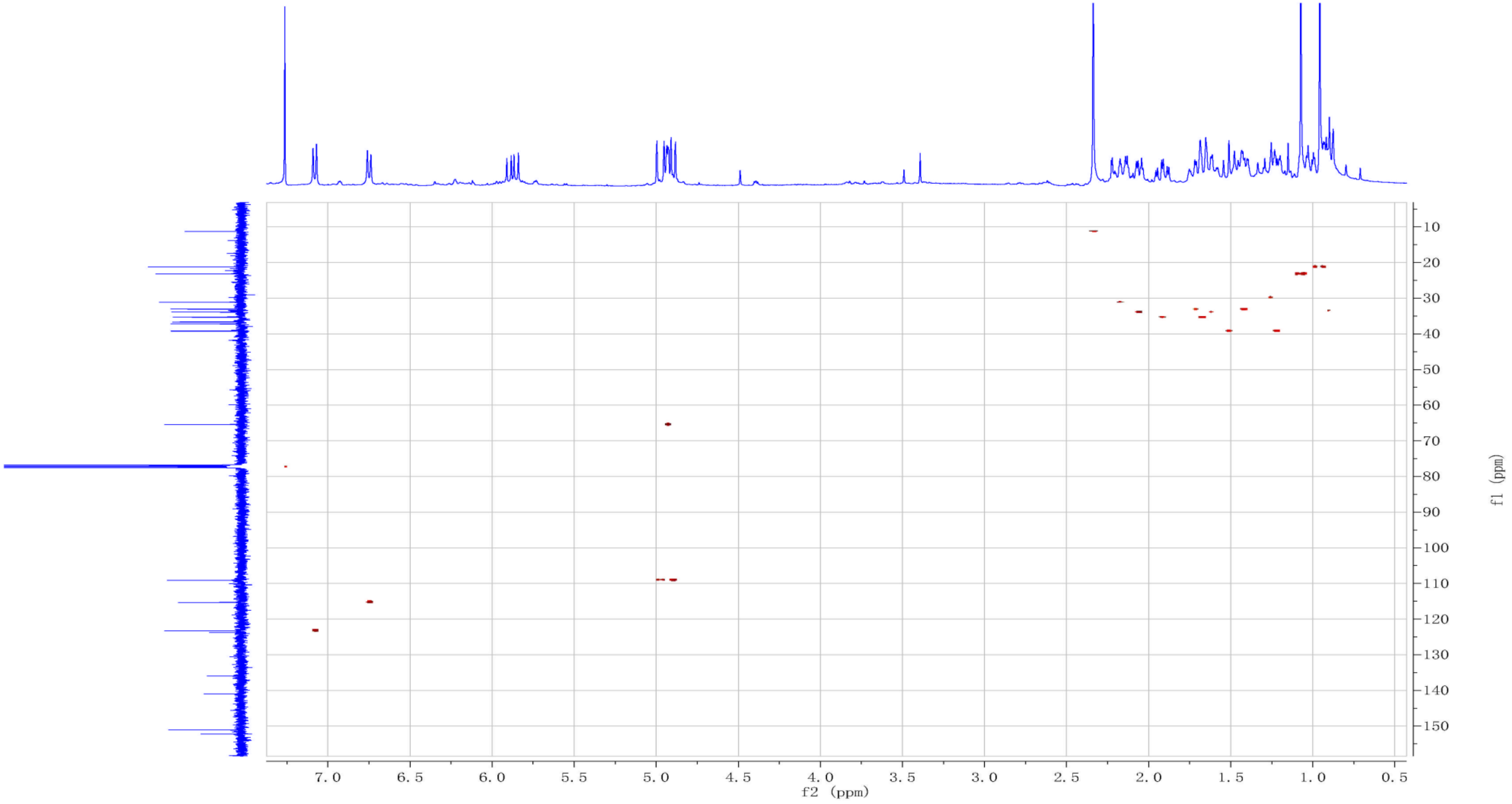

Figure S5. HSQC spectrum of compound **1**.

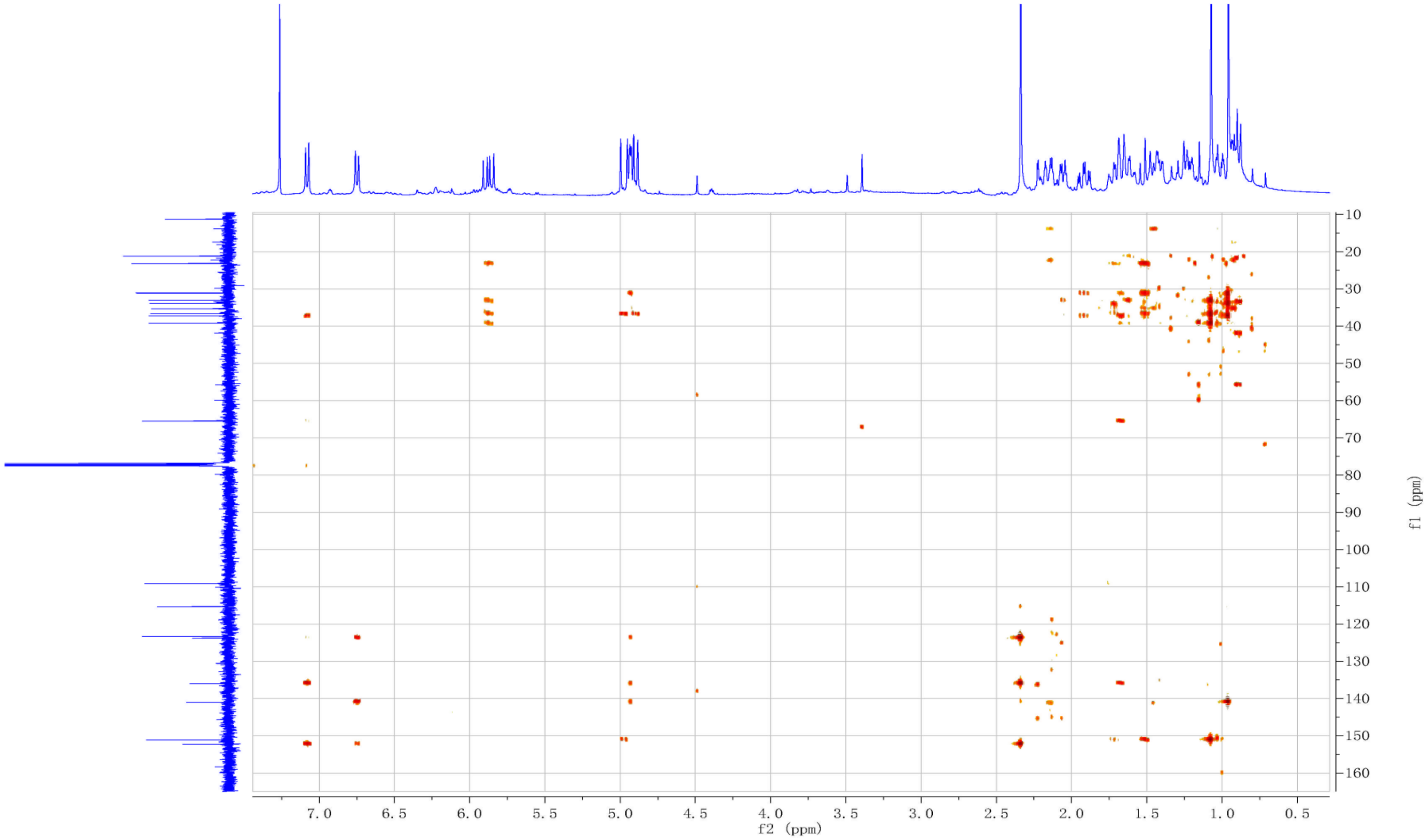

Figure S6. HMBC spectrum of compound **1**.

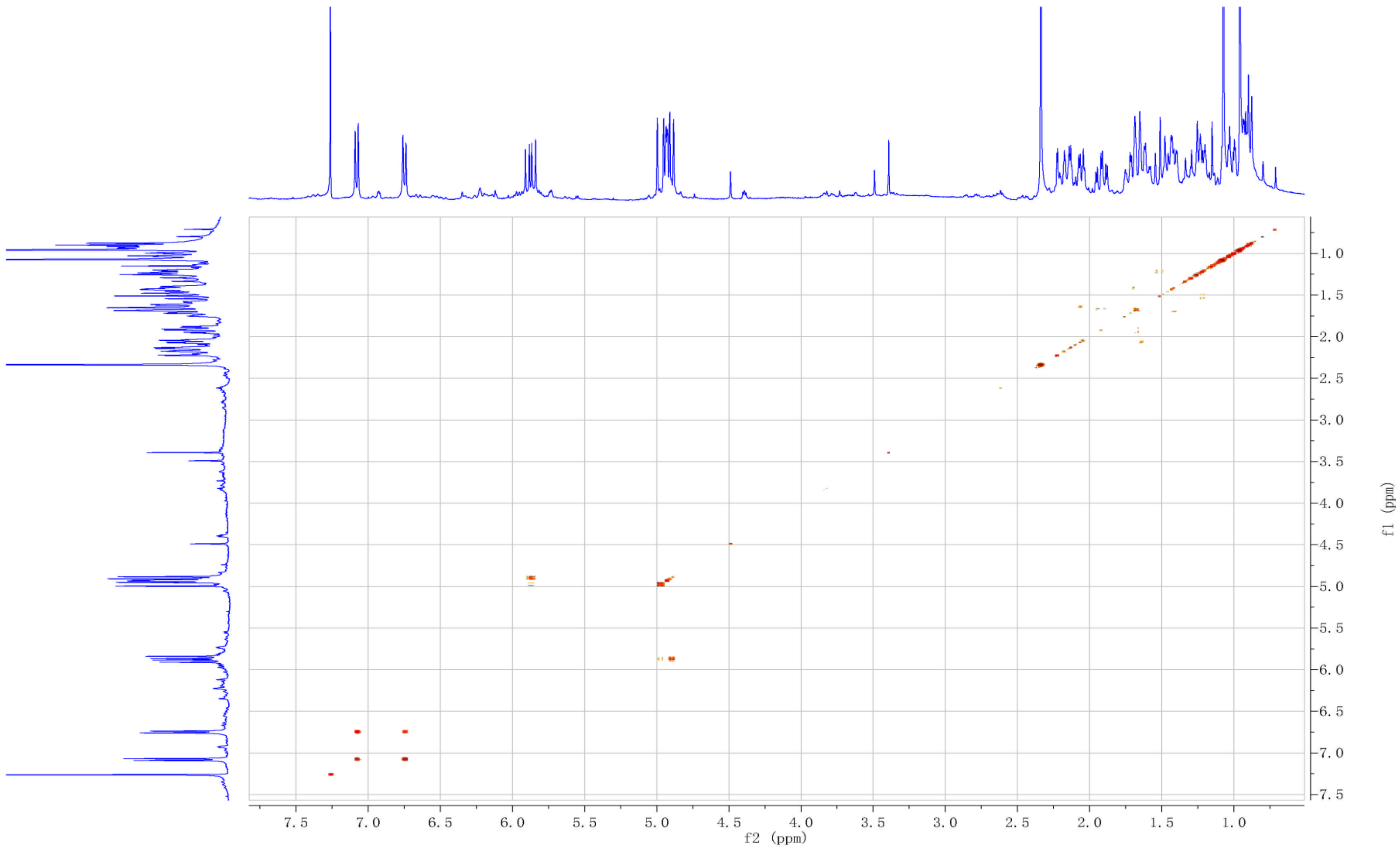

Figure S7. ^{1}H-^{1}H COSY spectrum of compound **1**.

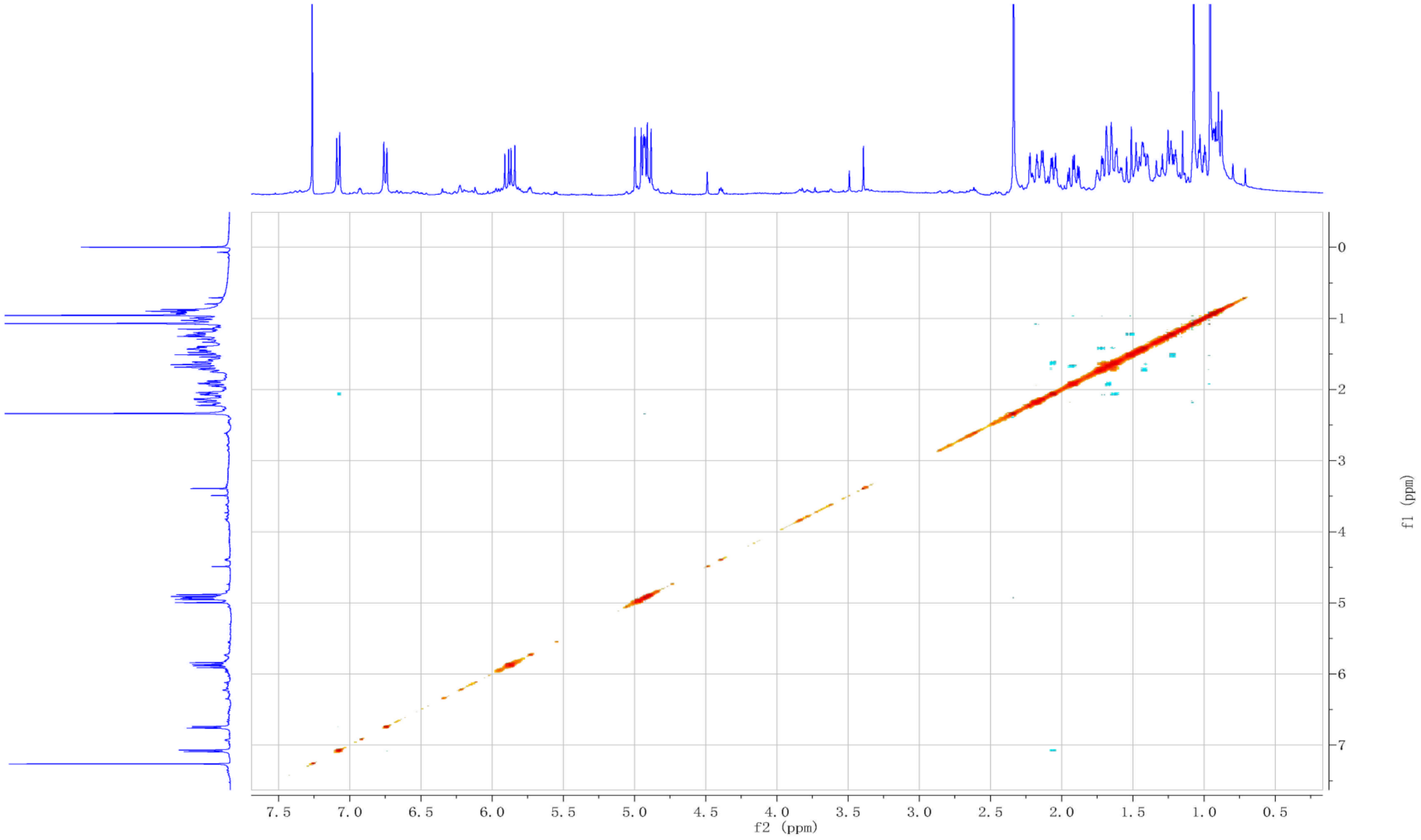

Figure S8. ROESY spectrum of compound **1**

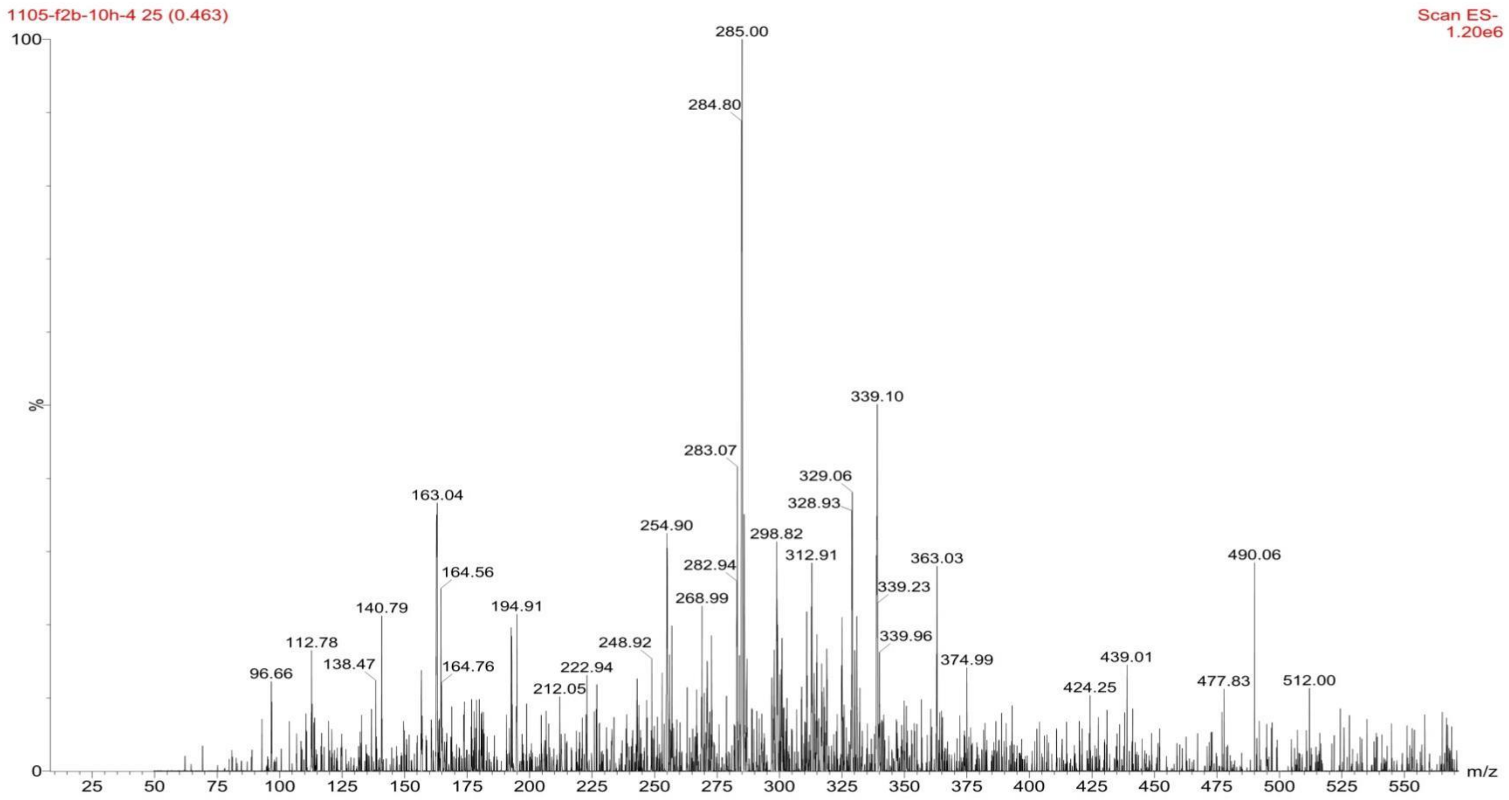

Figure S9. ESI-MS (negative mode) of compound **1**.

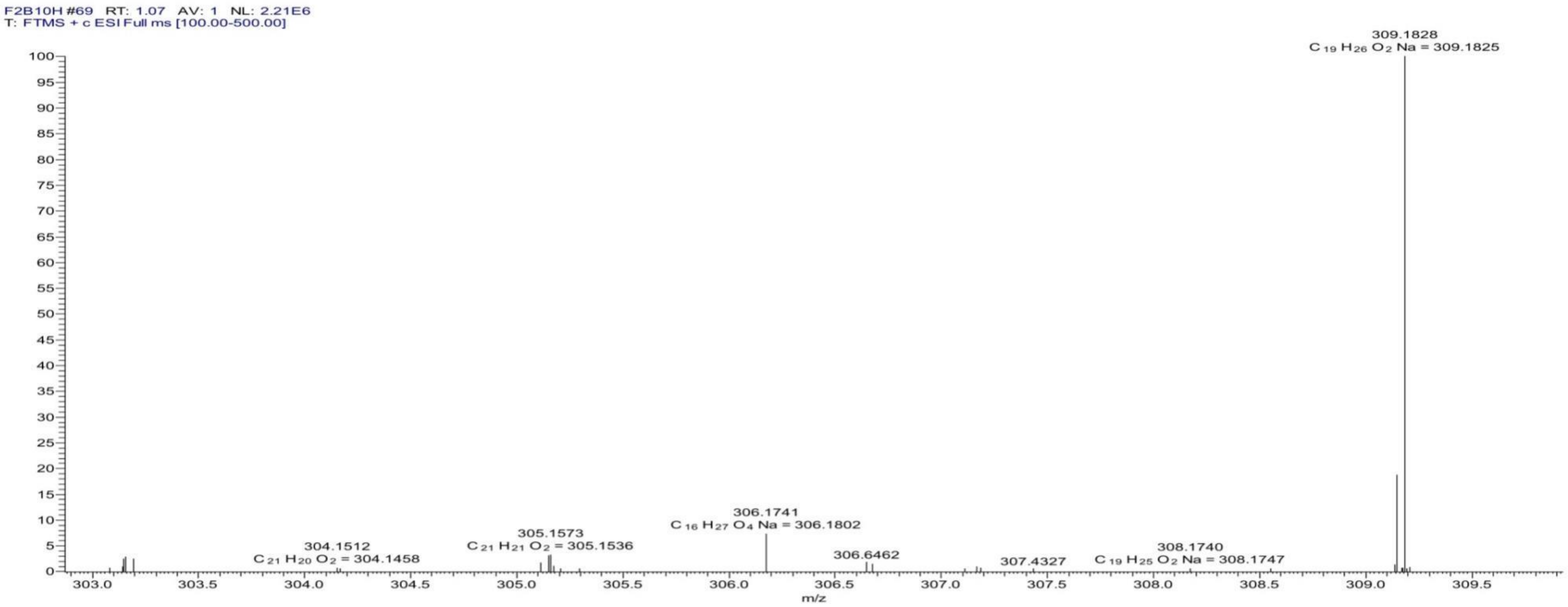

Figure S10. HR-ESI-MS (positive mode) of compound **1**

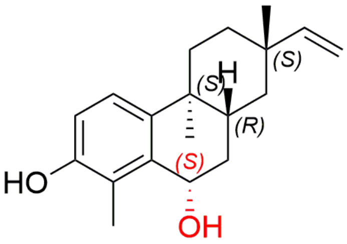

1a (6*S*,8*R*,9*S*,13*S*)

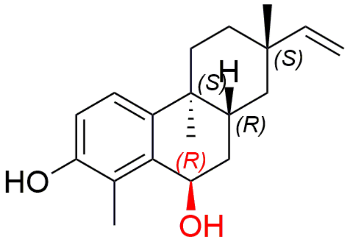

1b (6*R*,8*R*,9*S*,13*S*)

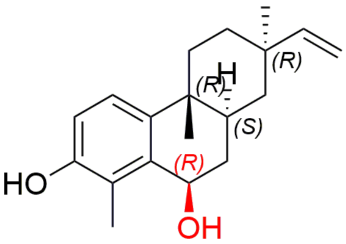

1a1 (6*R*,8*S*,9*R*,13*R*)

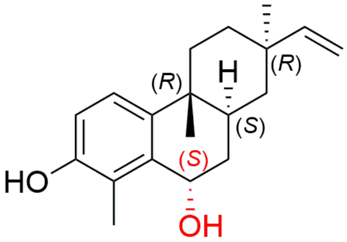

1b1 (6*S*,8*S*,9*R*,13*R*)

Figure S11. Four possible absolute configurations of compound **1**.

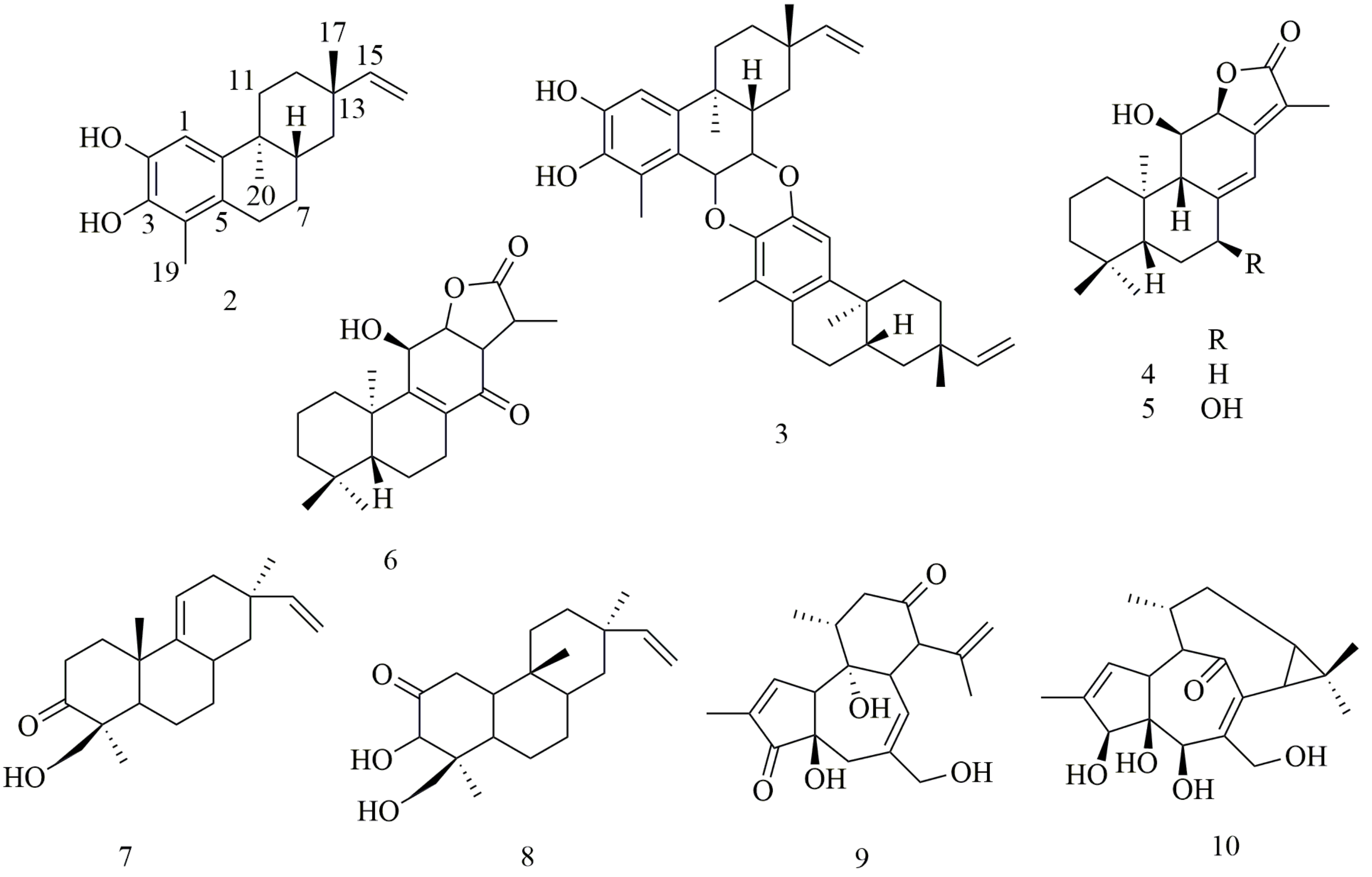

Figure S12. Structures of the known compounds **2-10**.

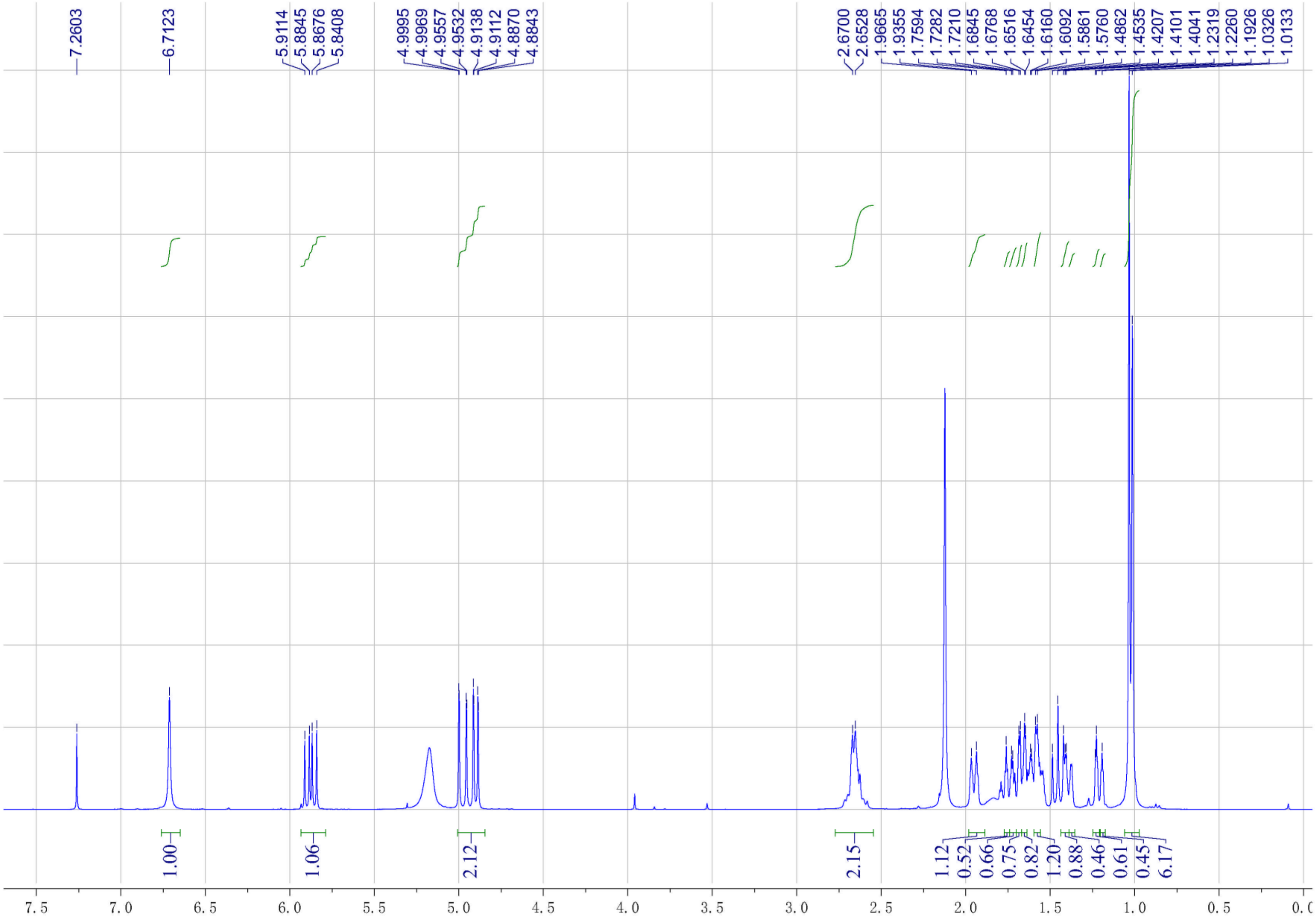

Figure S13. ^{1}H NMR spectrum ($CDCl_3$, 400MHz) of compound **2**.

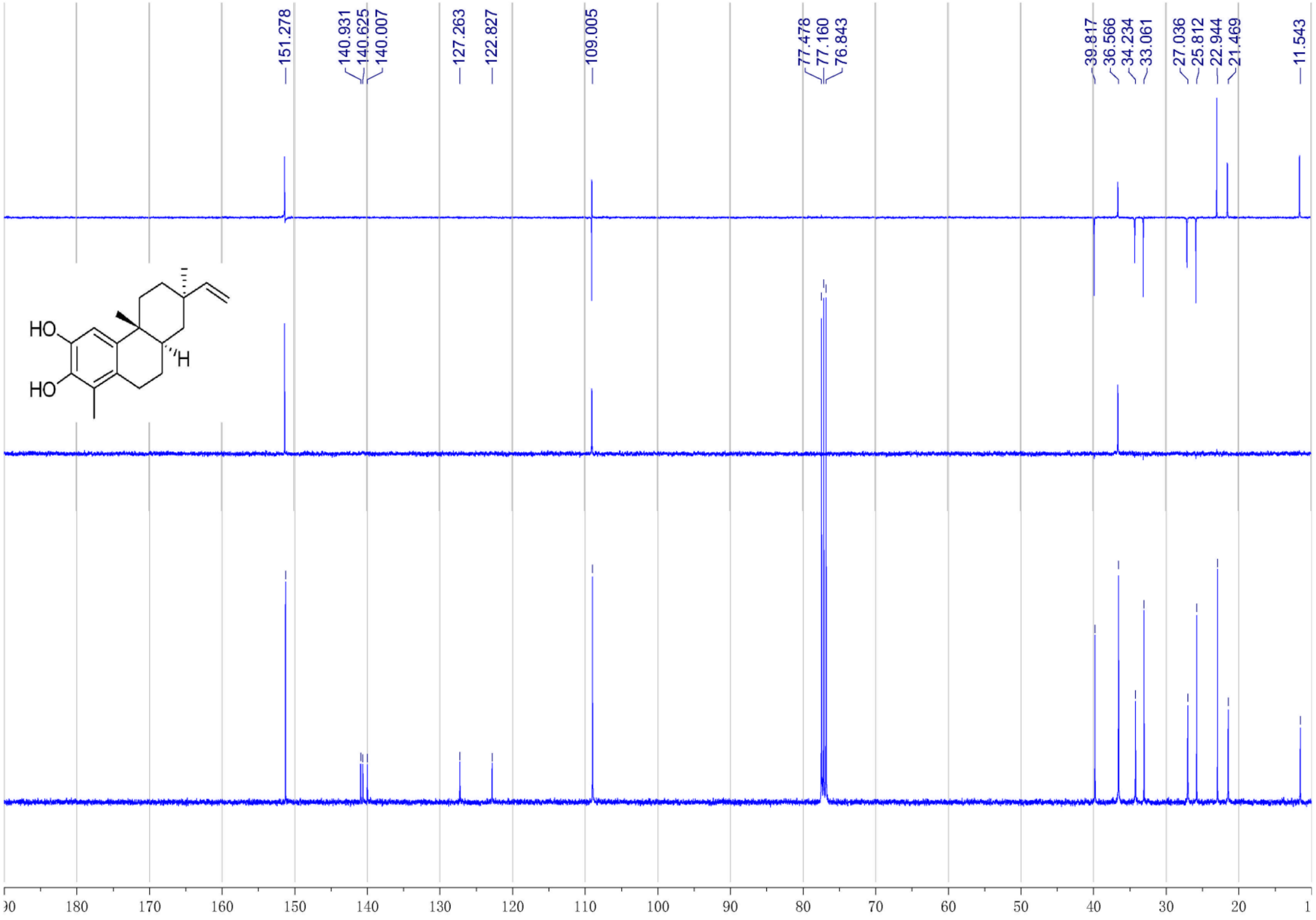

Figure S14. ^{13}C and DEPT spectra ($CDCl_3$, 100MHz) of compound **2**.

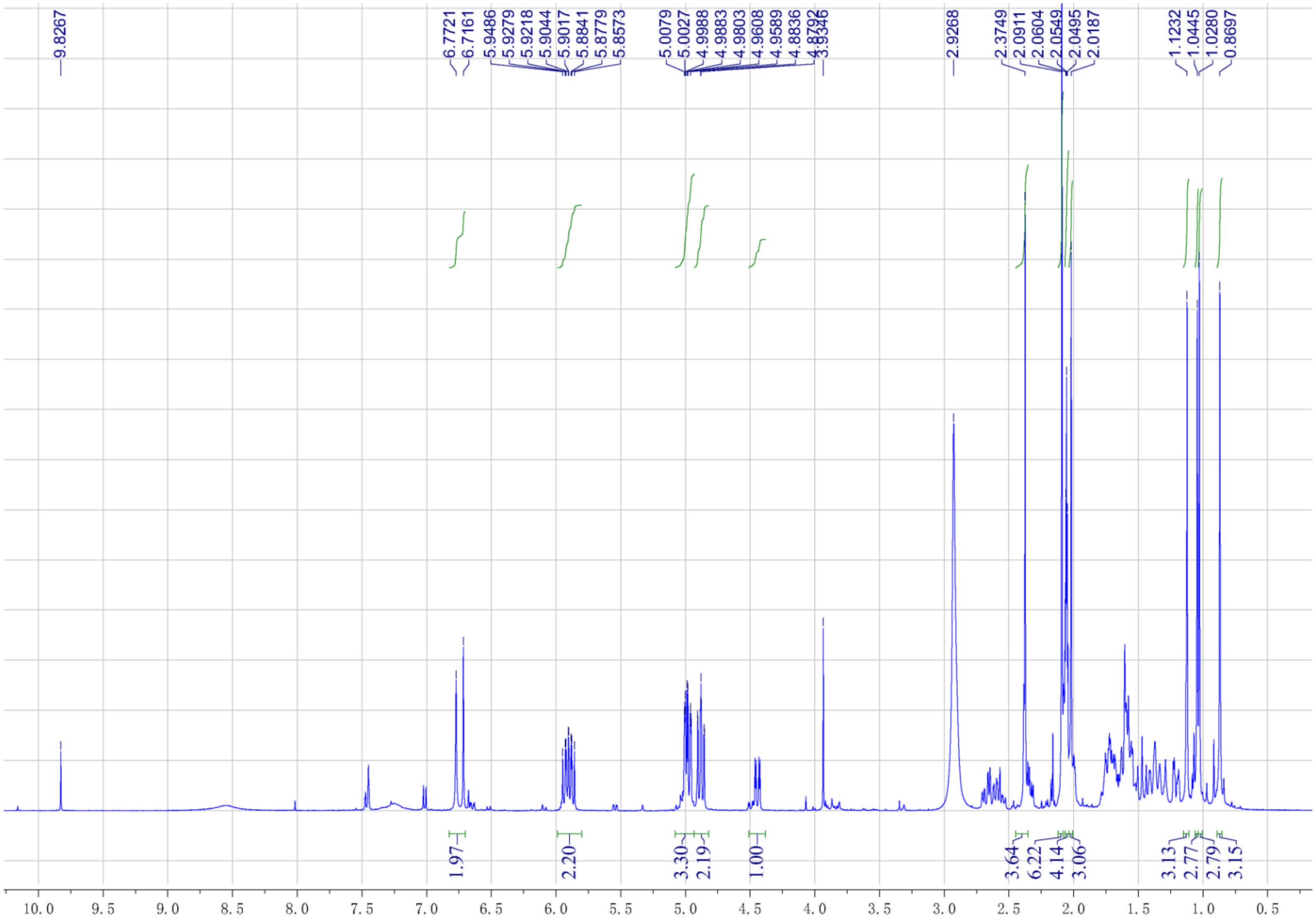

Figure S15. ^{1}H NMR spectrum (CD_3COCD_3, 400MHz) of compound **3.**

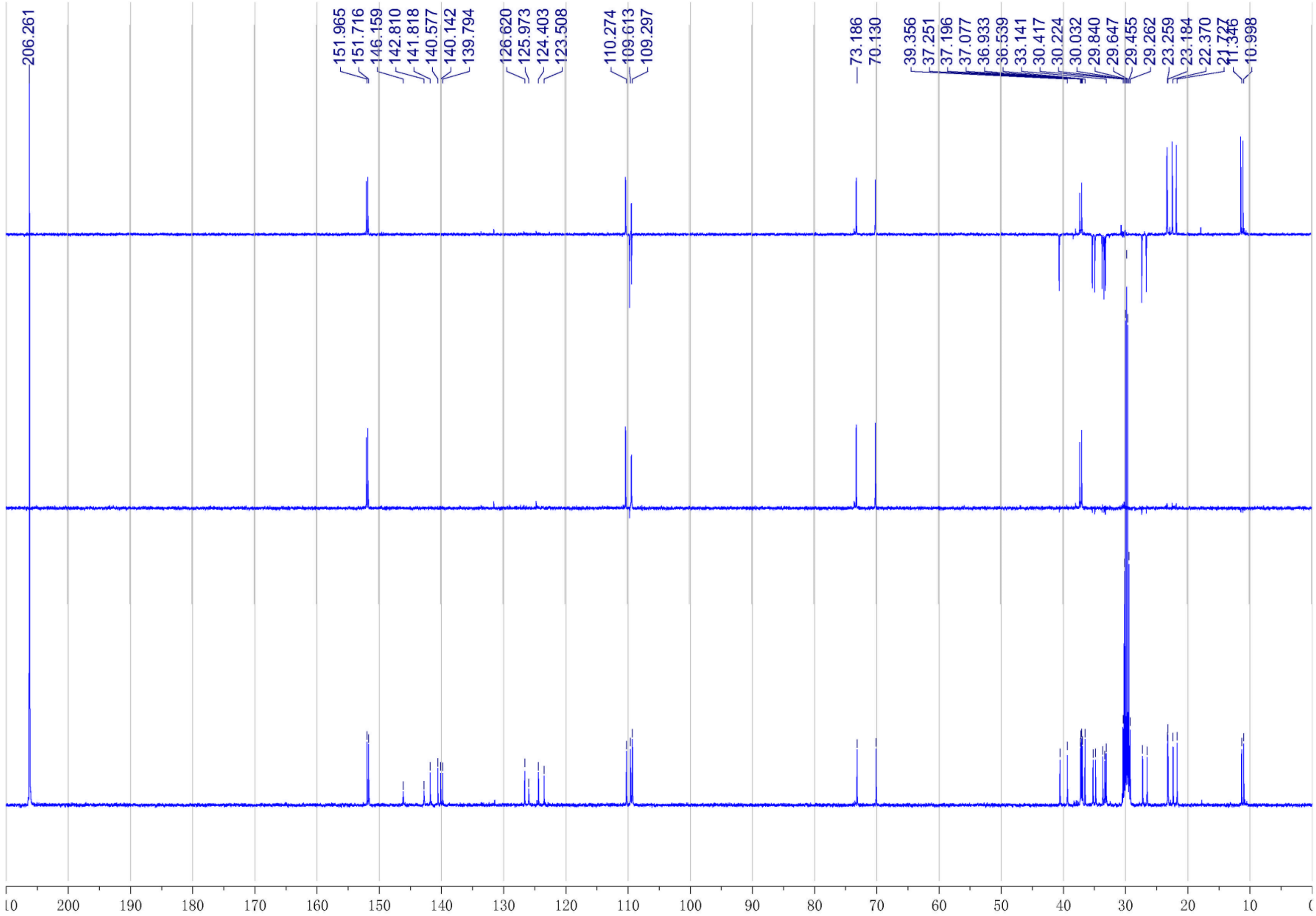

Figure S16. ^{13}C and DEPT spectra (CD_3COCD_3, 100MHz) of compound **3**.

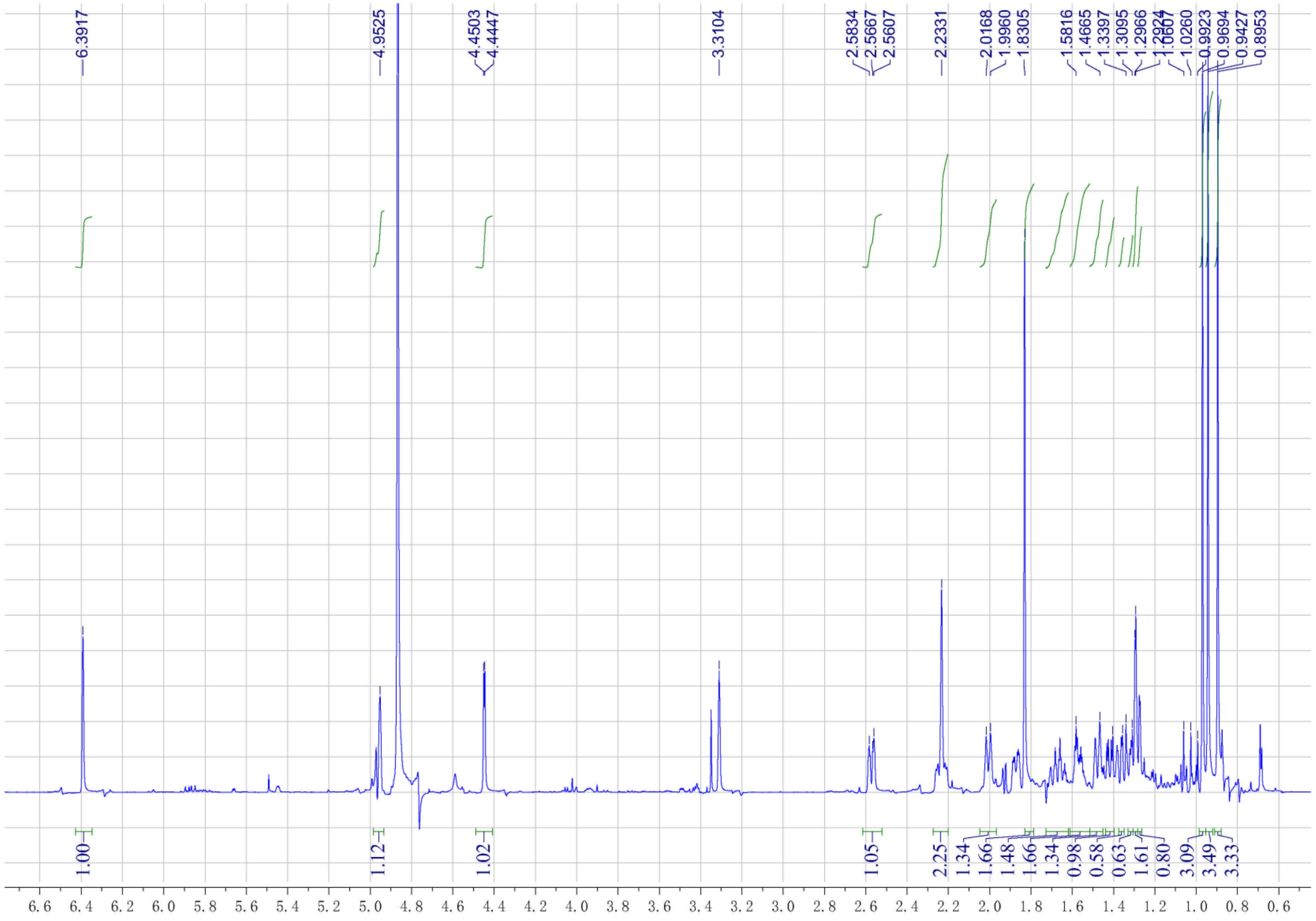

Figure S17. ^{1}H NMR spectrum (CD_3OD, 600MHz) of compound **4**.

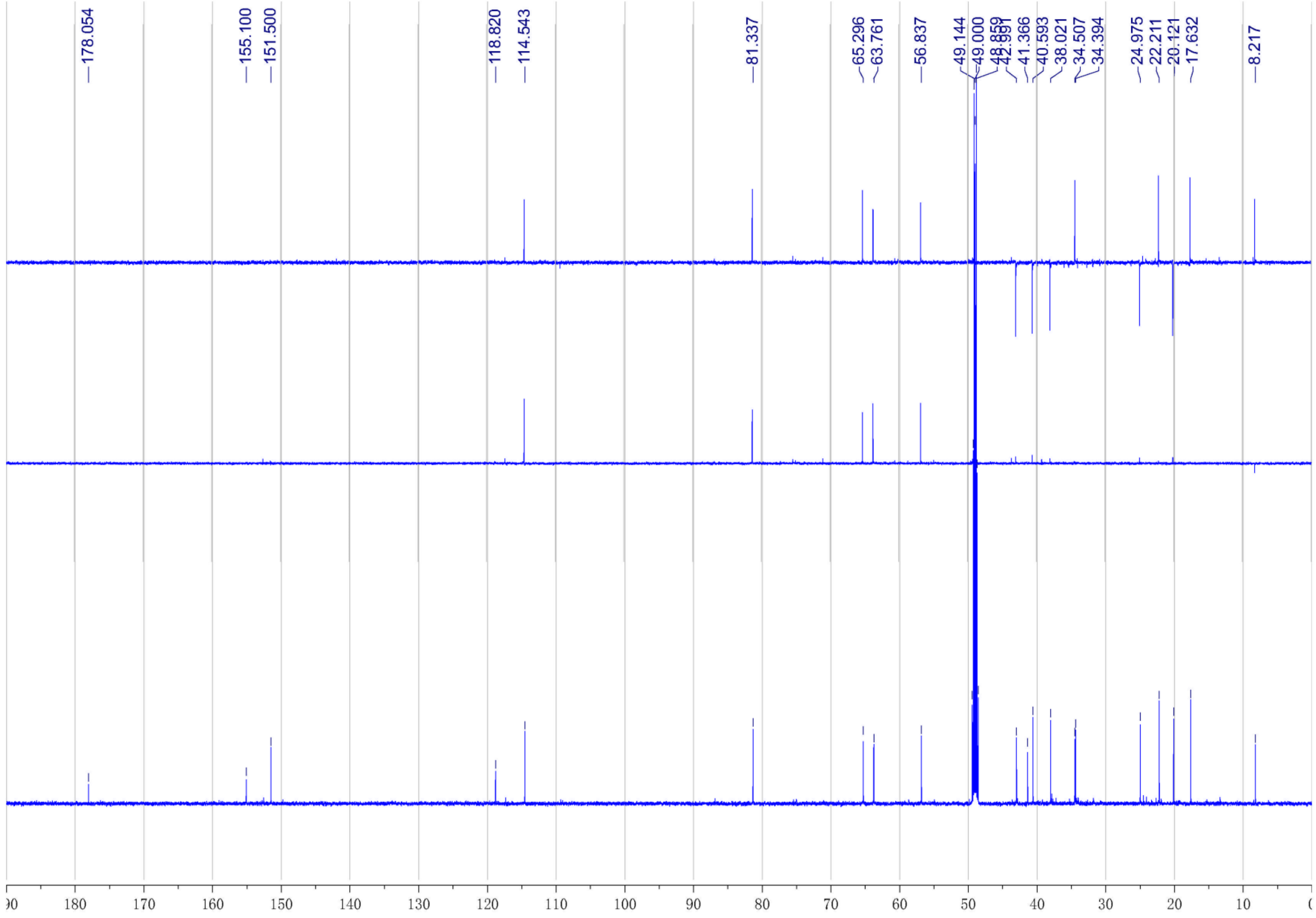

Figure S18. ^{13}C and DEPT spectra (CD_3OD, 150MHz) of compound **4**.

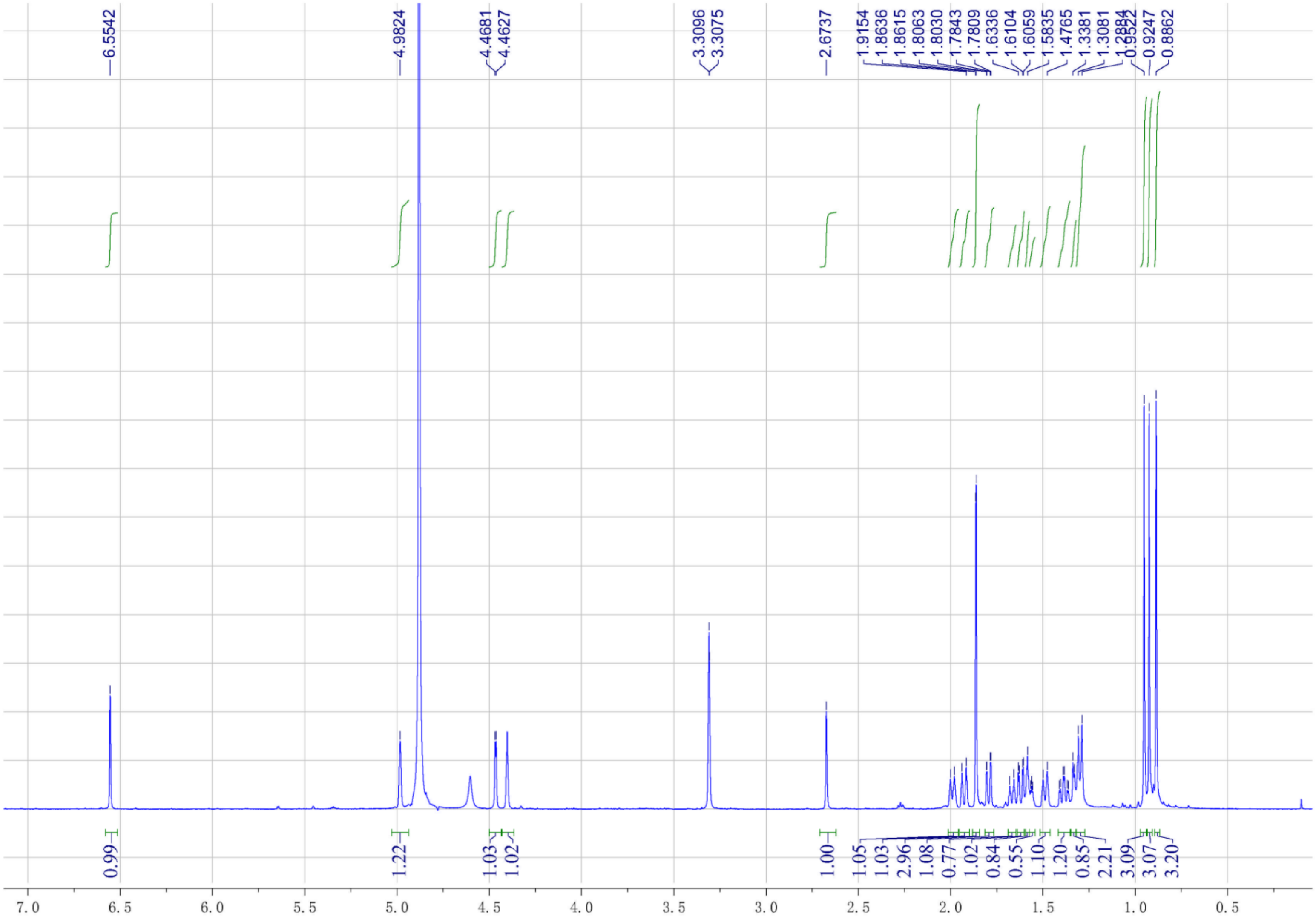

Figure S19. ^{1}H NMR spectrum (CD_3OD, 600MHz) of compound **5**.

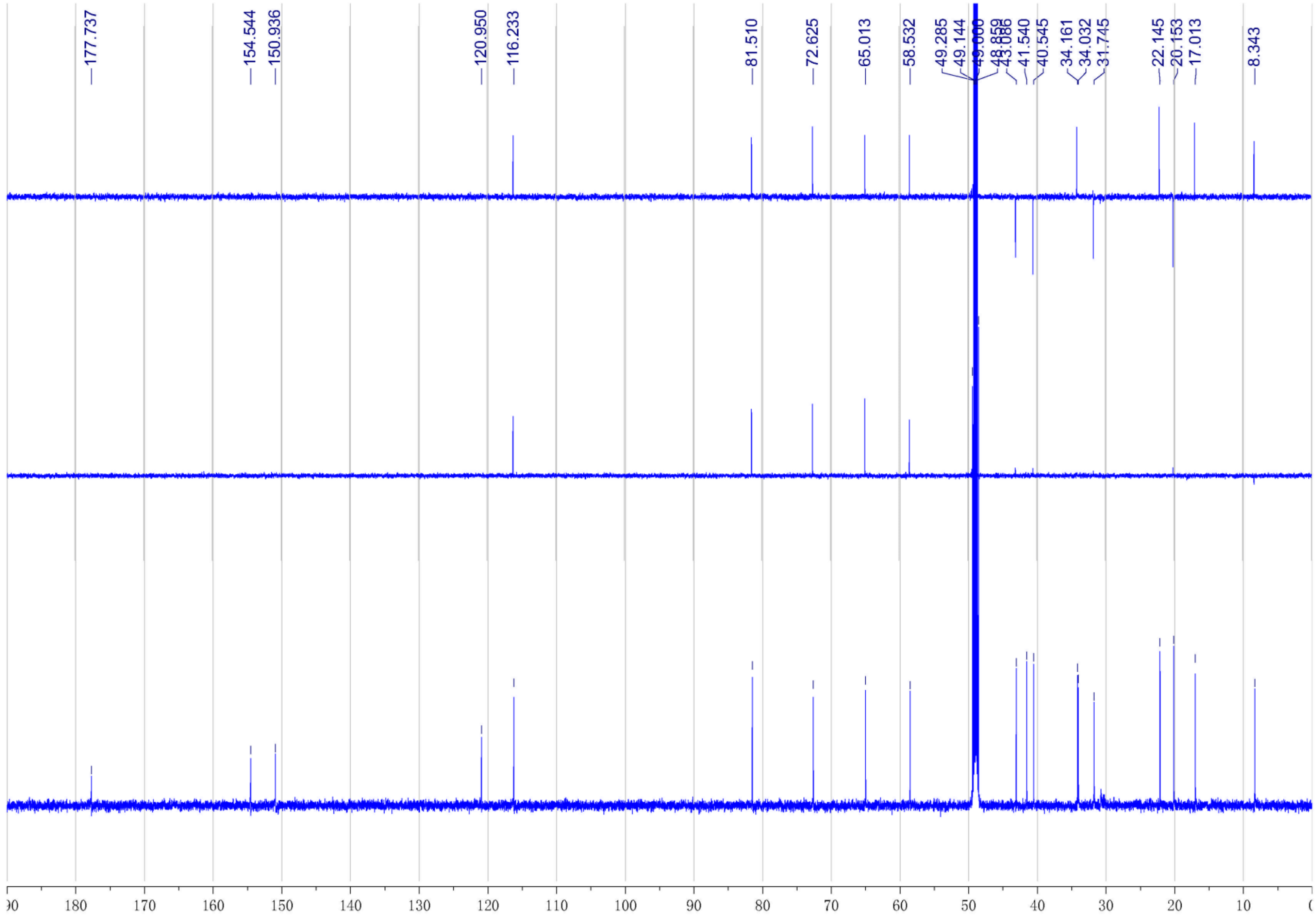

Figure S20. ^{13}C and DEPT spectra (CD_3OD, 150MHz) of compound **5**.

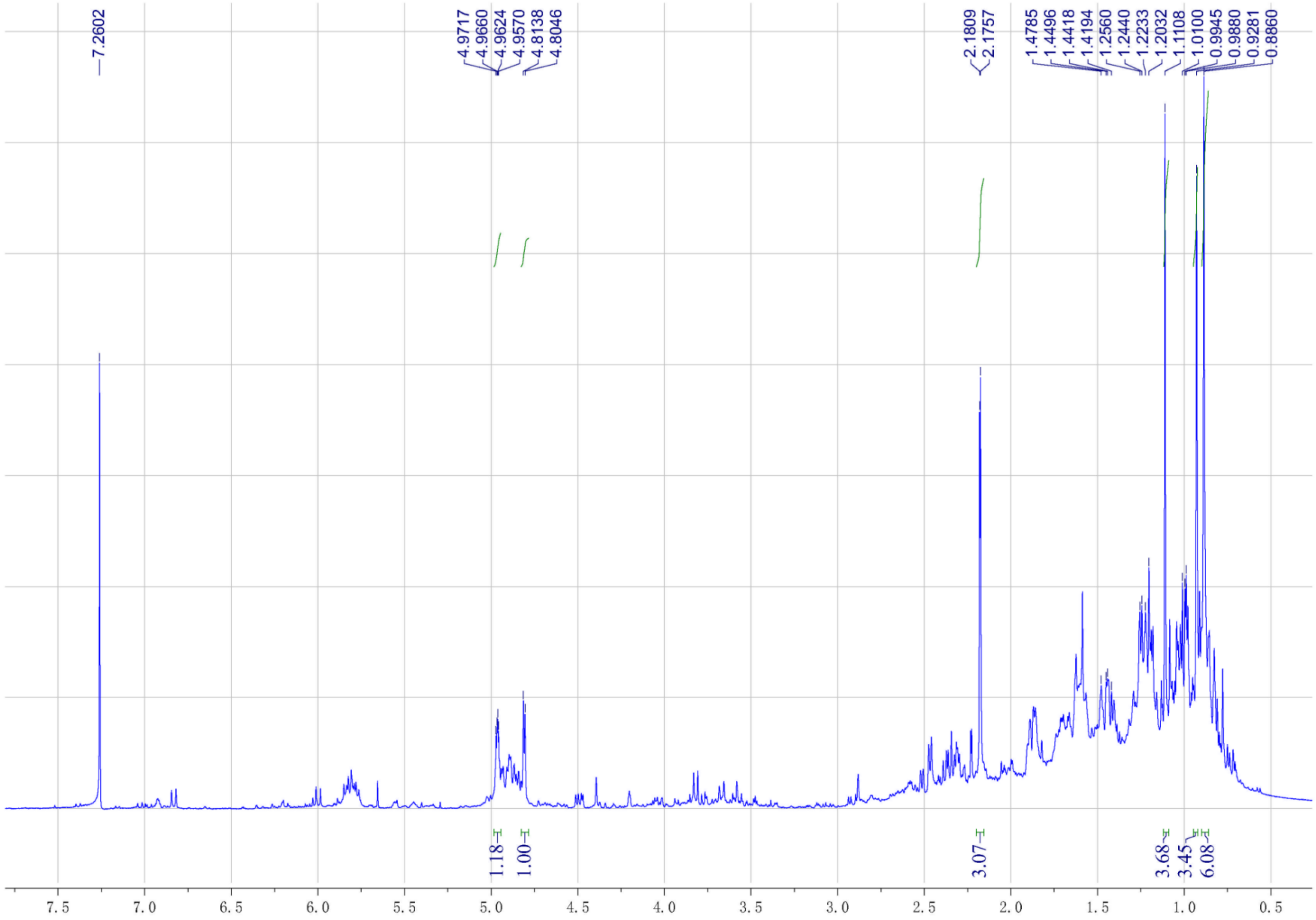

Figure S21. ^{1}H NMR spectrum ($CDCl_3$, 400MHz) of compound **6**

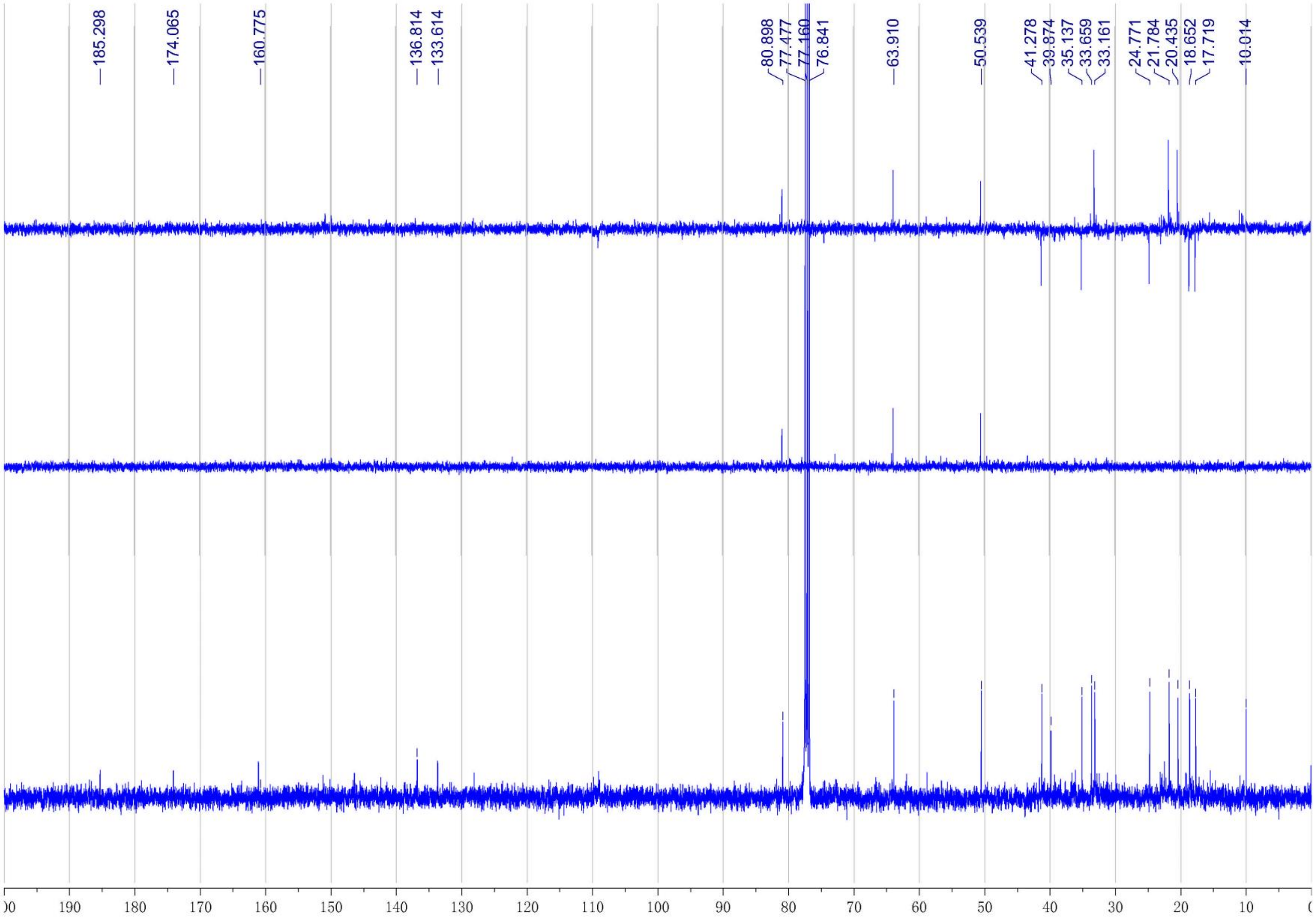

Figure S22. ^{13}C and DEPT spectra ($CDCl_3$, 100MHz) of compound **6**.

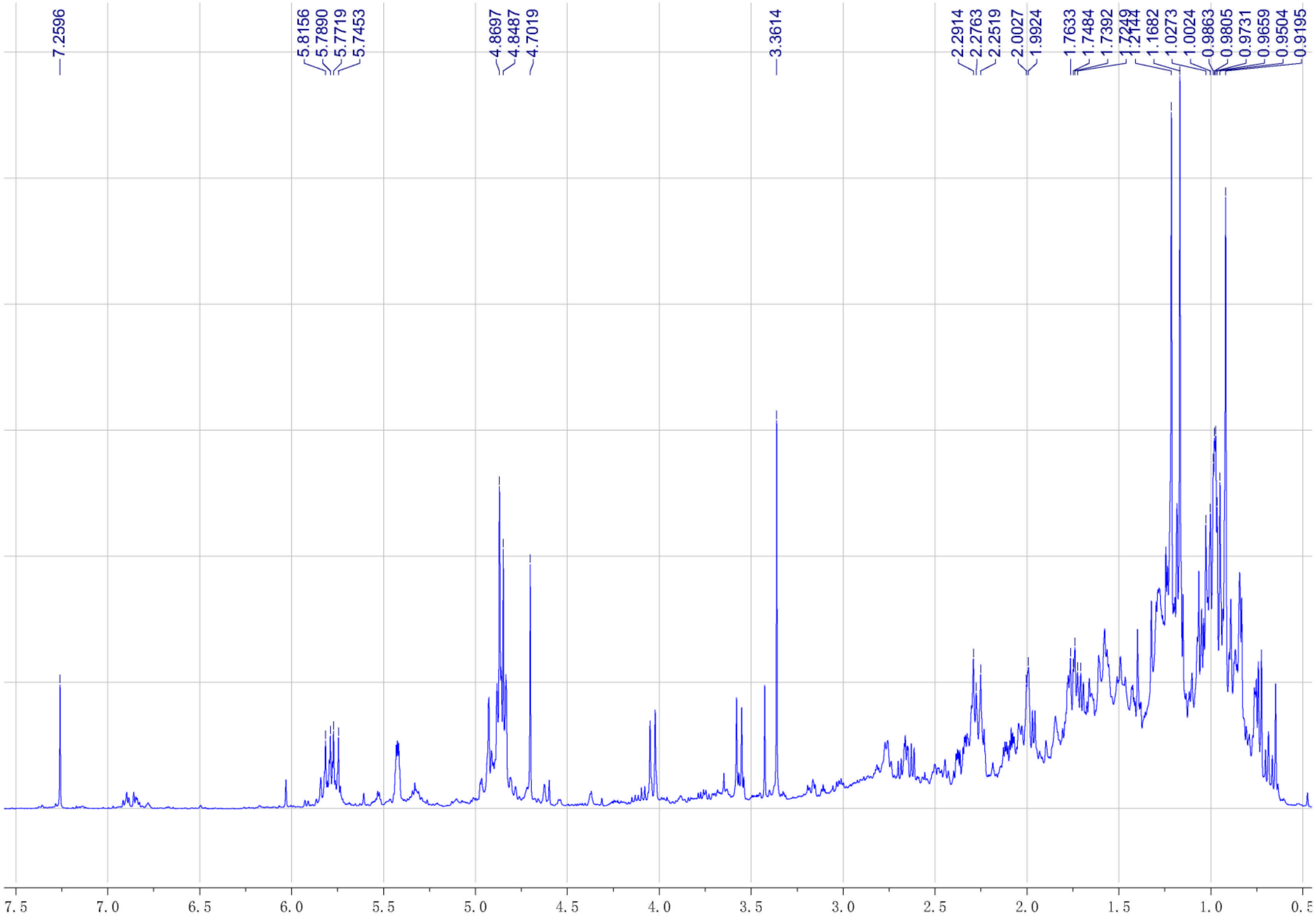

Figure S23. ^{1}H NMR spectrum ($CDCl_3$, 400MHz) of compound **7**.

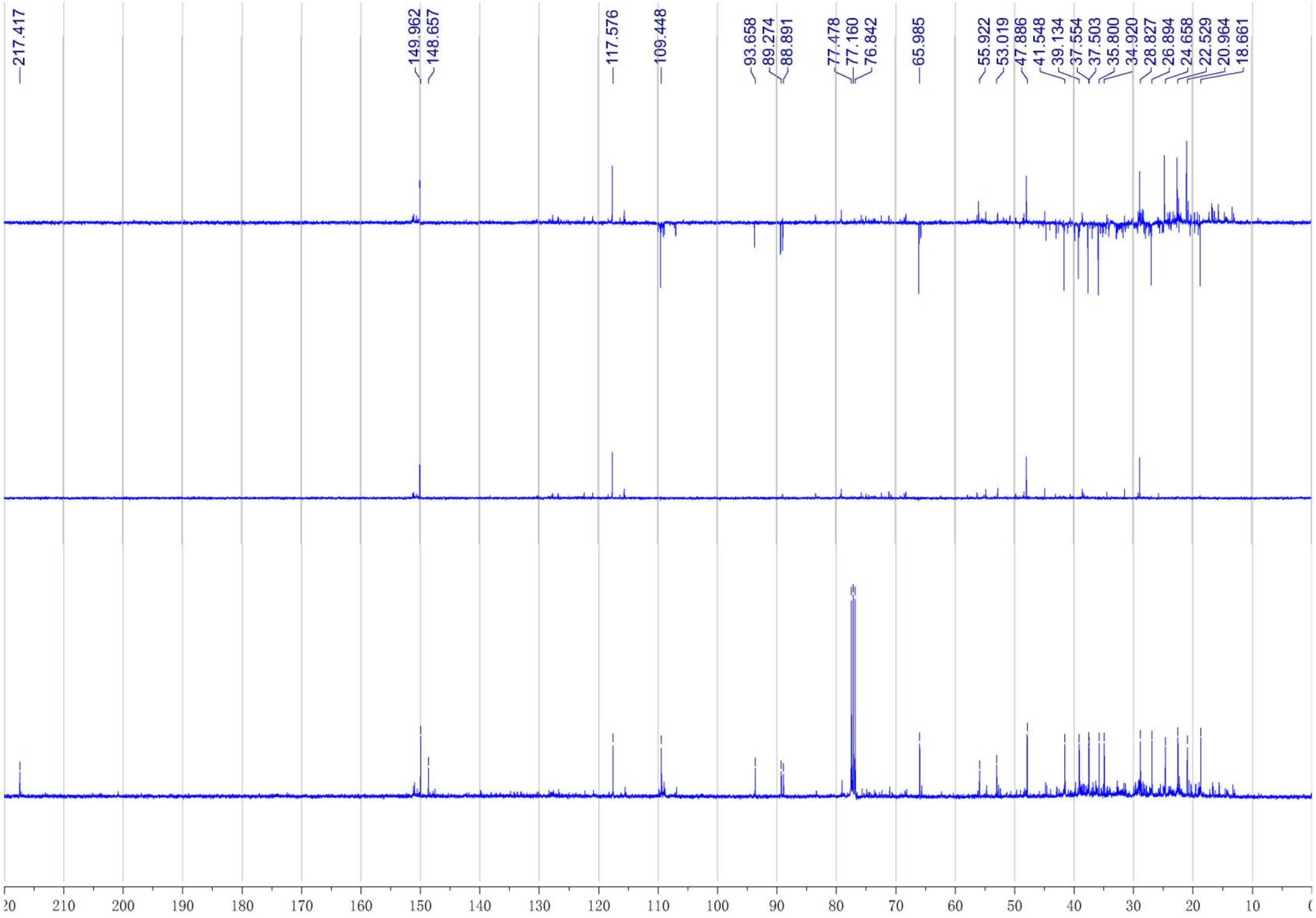

Figure S24. ^{13}C and DEPT spectra ($CDCl_3$, 100MHz) of compound **7**.

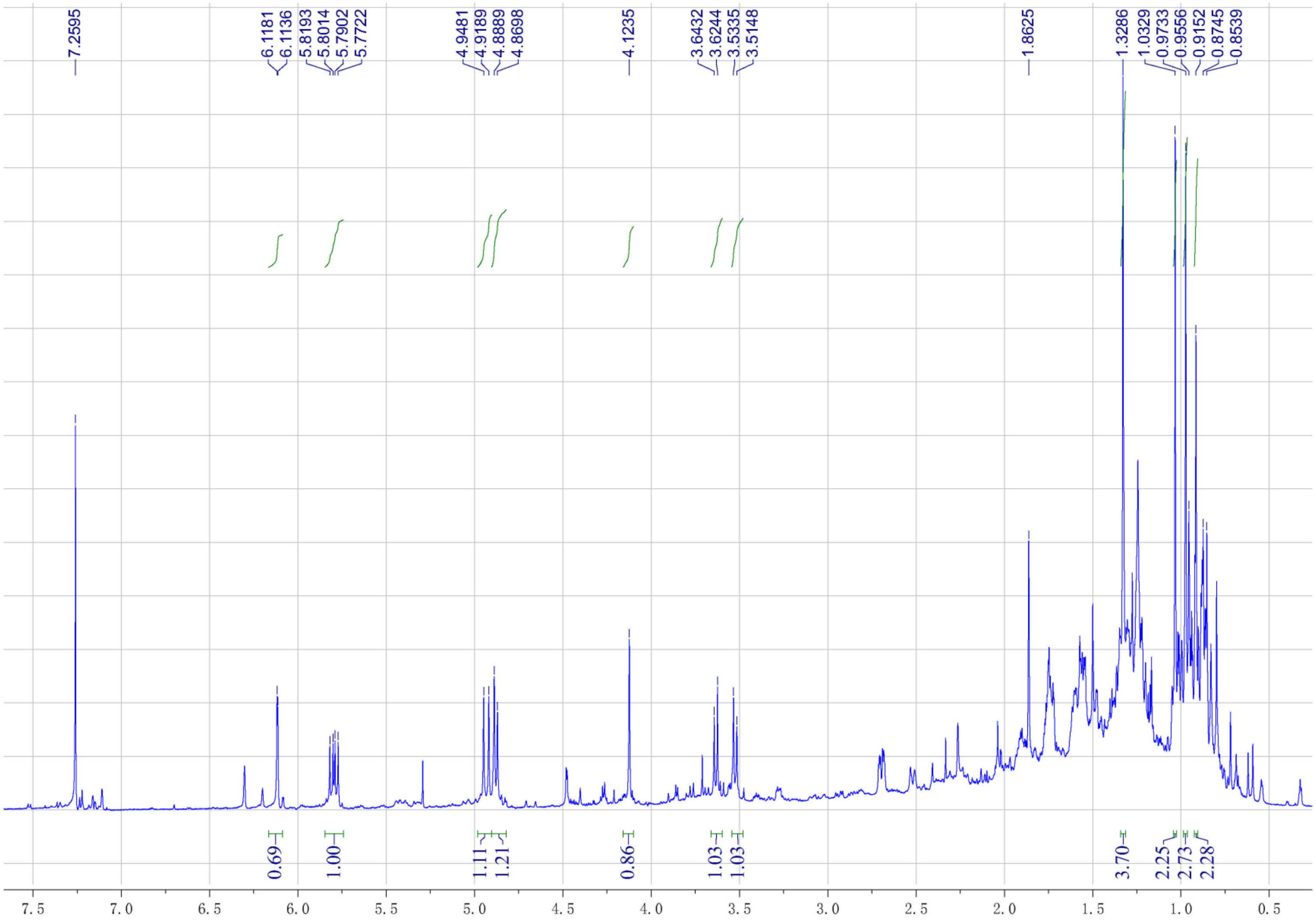

Figure S25. ^{1}H NMR spectrum (CDCl3, 600MHz) of compound **8**

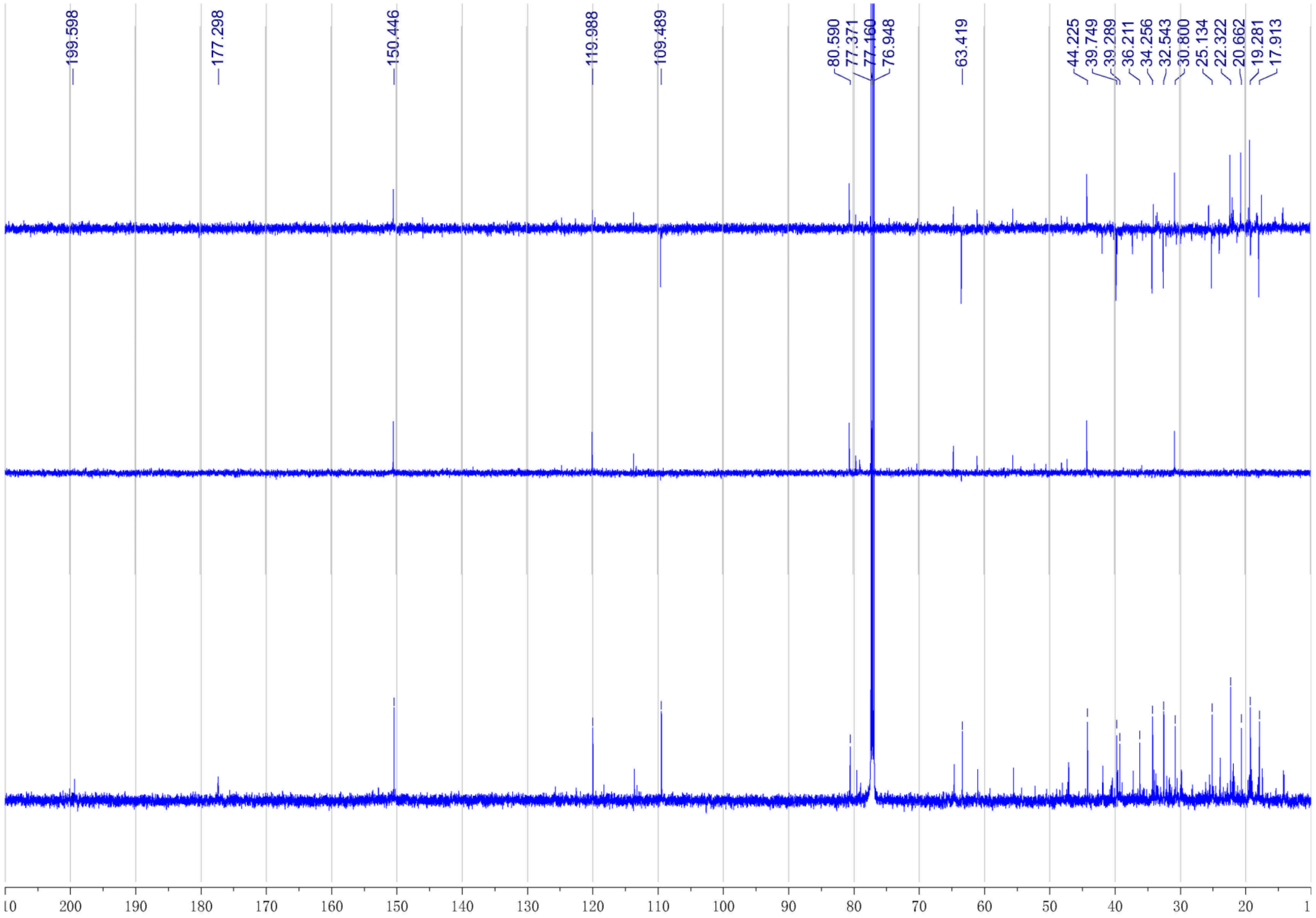

Figure S26. ^{13}C and DEPT spectra ($CDCl_3$, 150MHz) of compound **8**.

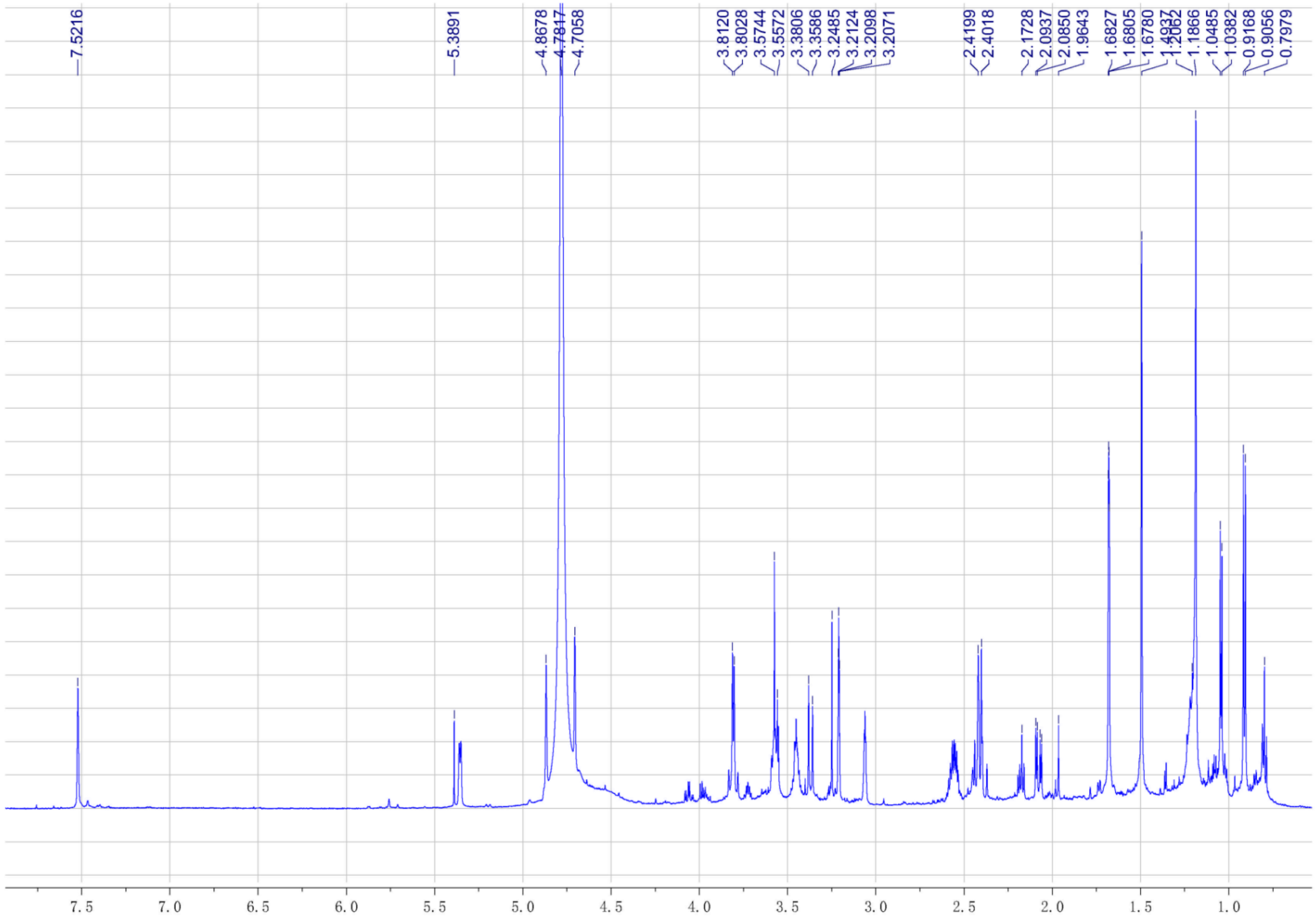

Figure S27. ^{1}H NMR spectrum ($CDCl_3$, 600MHz) of compound **9**.

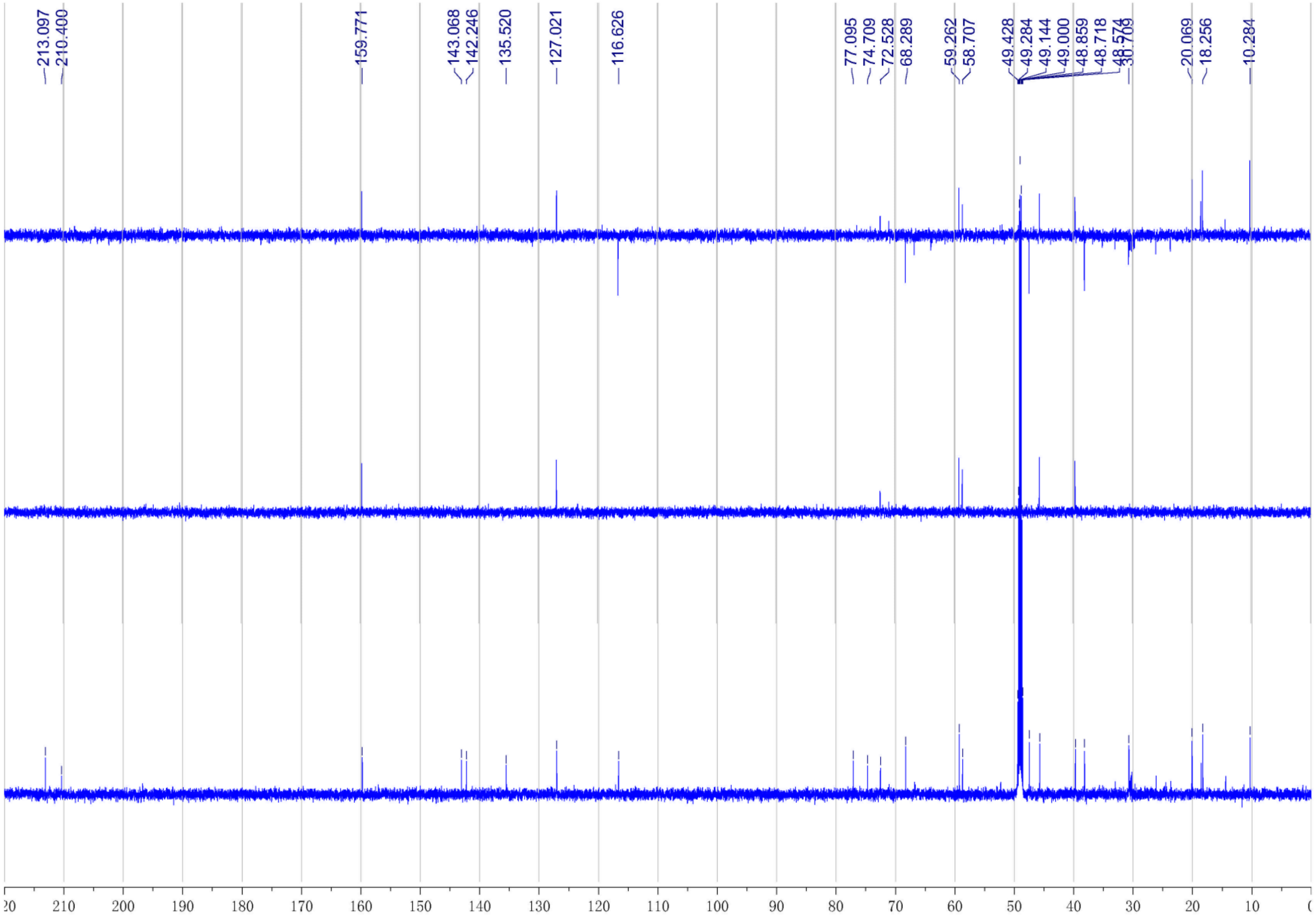

Figure S28. ^{13}C and DEPT spectra ($CDCl_3$, 150MHz) of compound **9**.

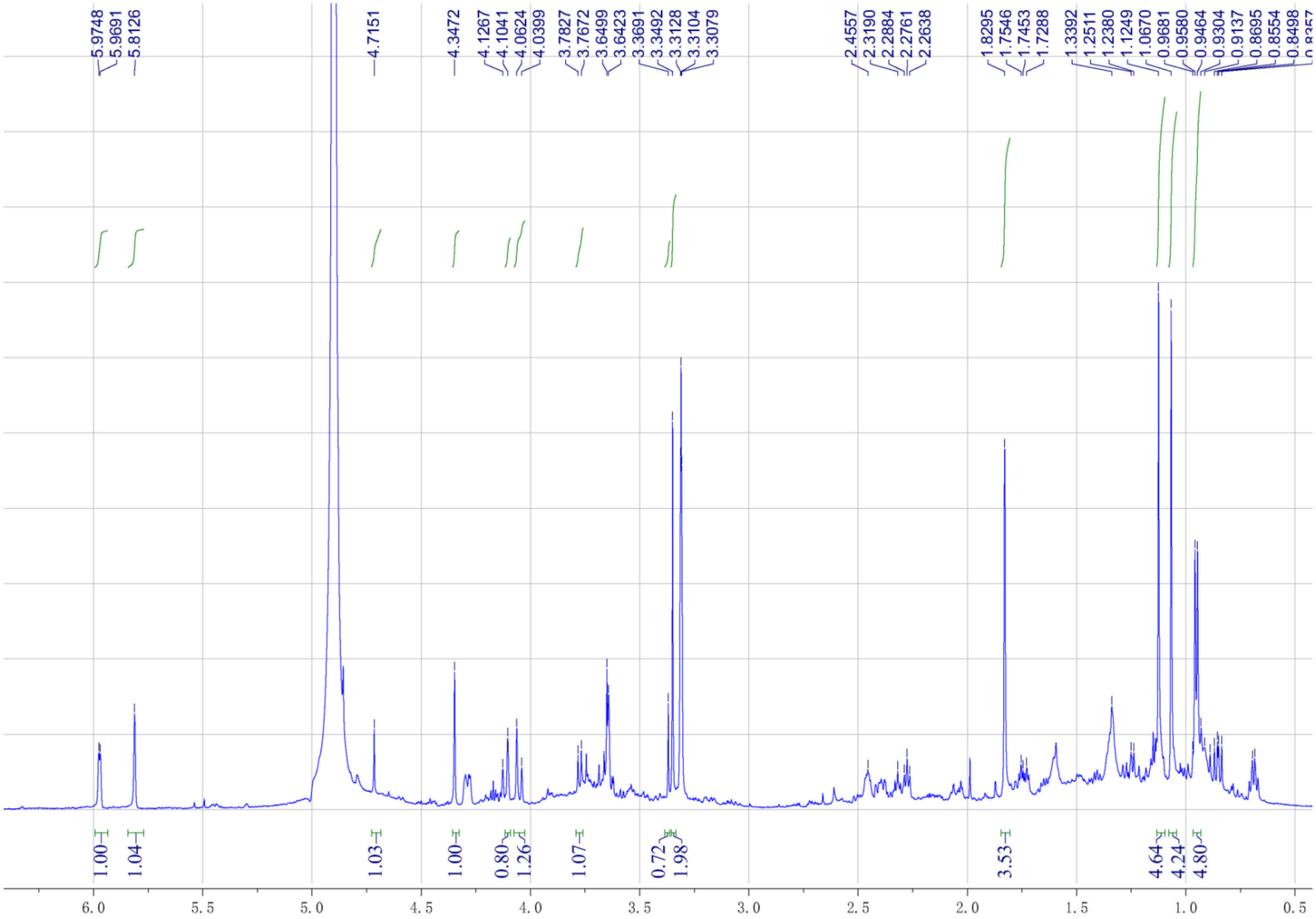

Figure S29. ^{1}H NMR spectrum (CDCl3, 600MHz) of compound **10**.

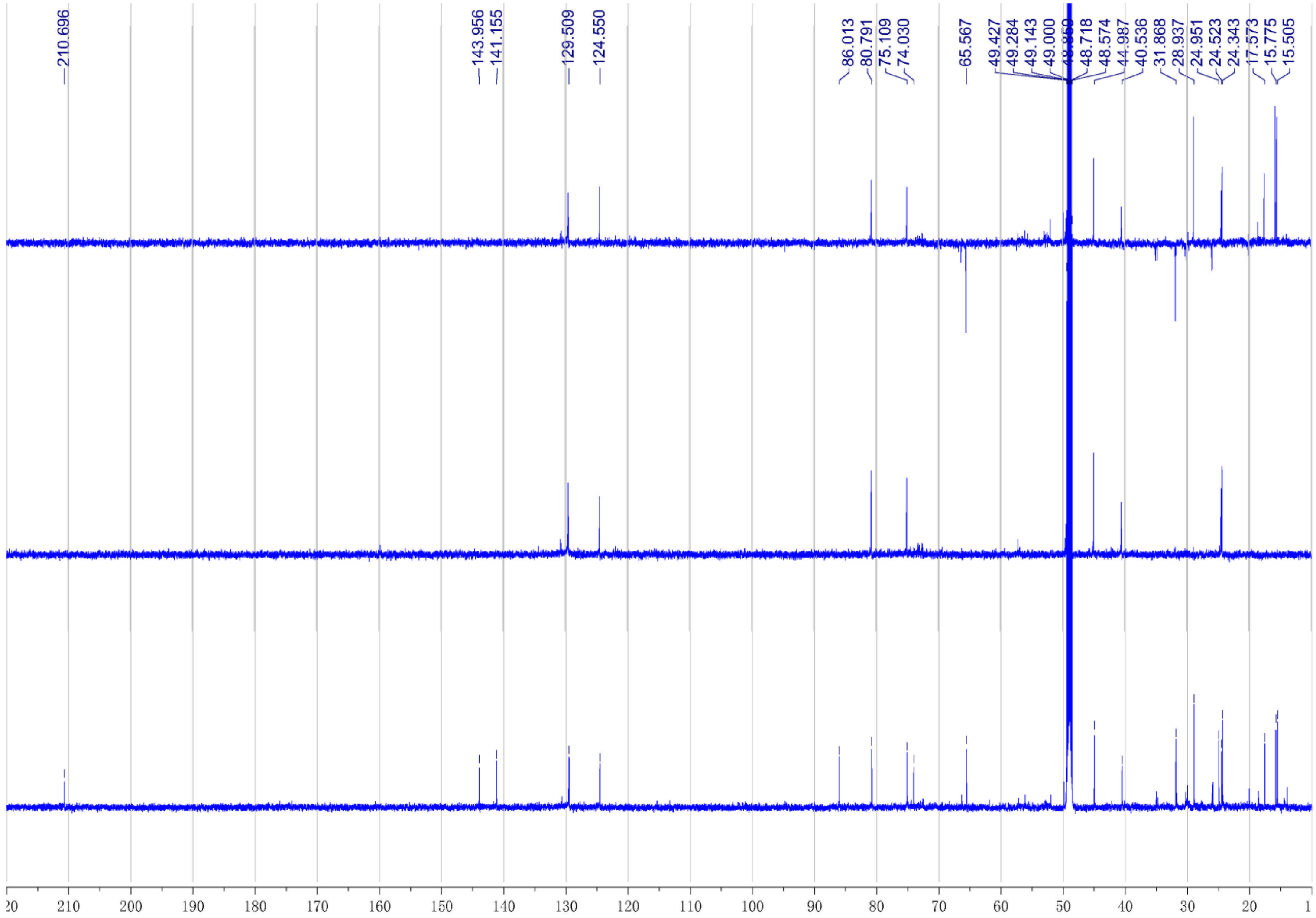

Figure S30. 13C and DEPT spectra (CDCl3, 150MHz) of compound 10.

Table S1. ^{1}H and ^{13}C NMR Data of Compound **1** and ebraphenol B (δ in ppm, data obtained in $CDCl_3$)

No.	1[a]		Ebraphenol B[b]	
	δ_H (mult, J, Hz)	δ_C	δ_H (mult, J, Hz)	δ_C
1	7.08 d (8.5)	123.3, CH	7.07 d (7.8)	123.0, CH
2	6.75 d (8.5)	115.3, CH	6.74 d (8.4)	115.2, CH
3		152.3, C		151.7, C
4		123.7, C		123.9, C
5		136.0, C		134.5, C
6	4.93 dd (6.0, 1.5)	65.5, CH	4.27 dd (3.6,1.2)	74.3, CH
7	1.91 dt (13.8, 4.2)<br>1.67 s (br.)	35.3, CH_2	1.82 dt (14.4,1.2)<br>1.65 m	28.8, CH_2
8	2.17 m	31.1, CH	2.14 m	31.1, CH
9		36.7, C		36.9, C
10		141.0, C		141.2, C
11	2.06 m<br>1.62 m	33.9, CH_2	2.03 dt (12.6,3.0)<br>1.60 m	33.8, CH2
12	1.71 m<br>1.41 m	33.1, CH_2	1.69 m<br>1.39 m	32.9, CH_2
13		37.2, C		36.6, C
14	1.50 m<br>1.21 m	39.2, CH_2		39.3, CH_2
15	5.87 dd (17.5, 10.7)	151.1, CH	5.88 dd (17.4, 10.2)	151.2, CH
16	4.97 dd (17.5, 1.2)<br>4.89 dd (10.7, 1.3)	109.1, CH_2	4.97 dd (17.4, 1.2)<br>4.89 dd (10.2, 1.2)	108.9, CH_2
17	1.07 s	23.2, CH_3	1.06 s	23.0, CH_3
19	2.34 s	11.3, CH_3	2.21 s	11.0, CH_3
20	0.96 s	21.2, CH_3	0.97 s	20.9, CH_3
-OCH_3			3.45 s	56.0, OCH_3

[a]*Data were measured at 100 MHz,* [b]*data were cited at 150MHz.*

Table S2. Key transitions, oscillator strengths, and rotatory strengths in the ECD spectrum of conformer **1b-1** at the B3LYP/6-31G (d, p) level of theory in MeOH with IEFPCM solvent model.

Num[a]	*Transition*[b]	*CI-coeff*[c]	*ΔE (eV)*[d]	*λ (nm)*[e]	*f*[f]	R_{vel}[g]	R_{len}[h]
1	77->80	-0.22509	4.7323	262.00	0.0633	1.2552	0.9274
	78->79	0.65387					
2	78->80	-0.26671	5.3152	233.26	0.0025	0.9	1.2856
	78->81	0.5653					
	78->83	-0.30808					
3	77->79	0.48167	5.4919	225.76	0.0090	-4.5138	-4.4442
	78->80	0.46103					
4	78->81	0.2393	5.6616	218.99	0.0033	0.5684	0.5208
	78->83	0.53524					
	78->86	-0.23029					
5	78->82	0.62423	5.7500	215.62	0.0003	0.3376	0.3776
	78->84	0.26478					
6	78->82	-0.31333	5.8121	213.32	0.0128	4.8007	4.8699
	78->84	0.52823					
7	77->79	0.23009	5.8577	211.66	0.1671	2.4422	2.559
	78->80	-0.23176					

	78->85	0.51807					
8	75->79	0.30047	5.9214	209.38	0.0622	4.0762	4.1504
	76->79	0.44762					
	78->85	-0.30153					
9	76->79	-0.38732	5.9888	207.03	0.1156	27.5929	28.1189
	77->80	-0.2487					
	77->81	0.3974					
10	75->79	0.37798	6.0475	205.02	0.1061	-20.4417	-20.1976
	76->79	-0.33008					
	77->81	-0.31808					

[a]Number of the excited states; [b]Transitions; [c]Configuration-interaction coefficient; [d]Excitation energy; [e]Wavelength; [f]Oscillator strength; [g]Rotatory strength in velocity form (10^{-40} cgs); [h]Rotatory strength in length form (10^{-40} cgs).

Table S3. Key transitions, oscillator strengths, and rotatory strengths in the ECD spectrum of conformer **1b-2** at the B3LYP/6-31+G(d,p) level of theory in MeOH with IEFPCM solvent model.

Num[a]	*Transition*[b]	*CI-coeff*[c]	*ΔE (eV)*[d]	*λ (nm)*[e]	*f*[f]	R_{vel}[g]	R_{len}[h]
1	77->80	-0.18889	4.7307	262.09	0.0626	0.7153	0.4778
	77->81	-0.16782					
	78->79	0.65404					
2	78->80	-0.35531	5.3150	233.27	0.0025	0.5188	0.9284
	78->81	0.33283					
	78->82	0.44934					
	78 -> 83	-0.22206					
3	77->79	0.48681	5.4938	225.68	0.0080	-3.7118	-3.6823
	78->80	0.38582					
	78->81	0.31725					
4	78->80	-0.27121	5.6209	220.58	0.0026	-4.554	-4.6319
	78->81	0.36405					
	78->82	-0.29305					
	78->83	0.34018					
	78->84	0.22006					
	78->86	-0.16845					

5	77->79	0.10398	5.7878	214.22	0.0347	14.6331	14.8708
	78->81	-0.21806					
	78->82	0.3575					
	78->83	0.48542					
	78->84	-0.14703					
	78->86	-0.20433					
6	78->81	-0.15648	5.8006	213.75	0.0046	-1.2064	-1.1872
	78->82	0.17029					
	78->84	0.57858					
	78->85	0.25885					
	78->87	0.14634					
7	75->79	0.16070	5.8864	210.63	0.1786	-5.8591	-5.9714
	76->79	-0.14716					
	77->79	0.22875					
	77->80	-0.10034					
	78->80	-0.24889					
	78->82	-0.18809					
	78->84	-0.22250					
	78->85	0.45781					
8	75->79	-0.32414	5.9303	209.07	0.0618	2.1852	2.2556

	76->79	0.35383					
	77->79	-0.16443					
	78->81	0.14284					
	78->85	0.40067					
9	75->79	0.18711	5.9950	206.81	0.0521	33.0786	34.0853
	76->79	-0.32373					
	77->79	-0.12647					
	77->80	0.35322					
	77->81	-0.25988					
	77->82	-0.29942					
	77->83	0.10590					
	78->85	0.14381					
10	76->79	0.31358	6.0829	203.82	0.2032	-27.4359	-27.7493
	77->79	0.22501					
	77->80	0.15158					
	77->81	-0.13958					
	77->82	-0.23372					
	78->80	-0.18502					
	78->81	-0.11414					

	78->83	0.11137					
	78->86	0.37718					
	78->90	0.11813					

[a]Number of the excited states; [b]Transitions; [c]Configuration-interaction coefficient; [d]Excitation energy; [e]Wavelength; [f]Oscillator strength; [g]Rotatory strength in velocity form (10^{-40} cgs); [h]Rotatory strength in length form (10^{-40} cgs).

Table S4. Key transitions, oscillator strengths, and rotatory strengths in the ECD spectrum of conformer **1b-3** at the B3LYP/6-31G (d, p) level of theory in MeOH with IEFPCM solvent model.

Num[a]	***Transition***[b]	***CI-coeff***[c]	***ΔE (eV)***[d]	***λ (nm)***[e]	***f***[f]	R_{vel}[g]	R_{len}[h]
1	77->80	0.25036	4.7685	260.01	0.0580	0.5235	0.1393
	78->79	0.64988					
2	78->80	0.16744	5.3294	232.64	0.0033	1.318	1.5357
	78->81	0.62768					
	78->83	0.24373					
3	77->79	-0.45749	5.4940	225.67	0.0181	-7.4573	-7.408
	78->80	0.51263					
	78->81	-0.14034					
4	78->80	-0.10329	5.6773	218.39	0.0017	1.8898	1.8615
	78->81	-0.19263					
	78->82	0.22689					
	78->83	0.56556					
	78->85	-0.11851					
	78->86	0.21643					
5	78->82	0.65224	5.7863	214.27	0.0005	-1.2515	-1.3147
	78->83	-0.20505					

	78->84	0.11023					
6	77->79	-0.17403	5.8586	211.63	0.1271	6.1222	6.1775
	78->80	-0.18490					
	78->84	0.60842					
7	75->79	-0.18794	5.8809	210.82	0.1475	17.0049	16.9946
	78->80	-0.19142					
	77->79	-0.29534					
	78->80	-0.21450					
	77->79	-0.25801					
	78->80	-0.16870					
	77->79	0.40255					
	78->80	0.11216					
8	75->79	0.15574	5.9469	208.48	0.0262	-4.5361	-4.4666
	76->79	0.27075					
	77->79	0.11193					
	77->80	0.11053					
	77->81	0.12475					
	78->84	0.21419					
	78->85	0.50026					
	78->86	0.13588					

9	76->79	0.52248	5.9994	206.66	0.0451	9.9651	10.1807
	77->80	-0.15764					
	77->81	-0.37456					
	77->83	-0.10579					
10	75->79	0.41144	6.0846	203.77	0.1012	-24.6681	-24.5262
	76->79	-0.29397					
	77->79	0.13895					
	77->81	-0.37783					
	77->83	-0.10910					
	78->80	0.12743					
	78->86	0.13416					

[a]Number of the excited states; [b]Transitions; [c]Configuration-interaction coefficient; [d]Excitation energy; [e]Wavelength; [f]Oscillator strength; [g]Rotatory strength in velocity form (10^{-40} cgs); [h]Rotatory strength in length form (10^{-40} cgs).

Table S5. Key transitions, oscillator strengths, and rotatory strengths in the ECD spectrum of conformer **1b-4** at the B3LYP/6-31G (d, p) level of theory in MeOH with IEFPCM solvent model.

Num[a]	***Transition***[b]	***CI-coeff***[c]	***ΔE (eV)***[d]	***λ (nm)***[e]	***f***[f]	R_{vel}[g]	R_{len}[h]
1	77->80	0.2503	4.7684	260.01	0.0580	0.5145	0.1324
	78->79	0.6499					
2	78->80	0.16785	5.3294	232.64	0.0033	1.3182	1.5374
	78->81	0.6277					
	78->83	0.24345					
3	77->79	-0.45744	5.4940	225.67	0.0181	-7.4645	-7.4141
	78->80	0.51259					
	78 -> 81	-0.14063					
4	78->80	-0.10334	5.6775	218.38	0.0017	1.9355	1.9074
	78->81	-0.19221					
	78->82	0.22697					
	78->83	0.56556					
	78->85	0.22697					
	78->86	0.56556					
5	78->82	0.6523	5.7864	214.27	0.0005	-1.2461	-1.3091
	78->83	-0.20514					
	78->84	0.10976					

6	77->79	-0.17479	5.8587	211.62	0.1278	6.1343	6.1884
	78->80	-0.18854					
	78->84	0.60796					
7	75->79	-0.18829	5.8809	210.82	0.1465	16.986	16.9768
	76->79	-0.19282					
	77->79	-0.29484					
	77->81	-0.21383					
	78->80	-0.25740					
	78->84	-0.17054					
	78->85	0.40199					
	78->88	0.11222					
8	75->79	0.15576	5.9469	208.49	0.0256	-4.5062	-4.4369
	76->79	0.27236					
	77->79	0.11082					
	77->80	0.10976					
	77->81	0.12278					
	78->84	0.21397					
	78->85	0.50061					
	78->86	0.13569					

9	76->79	0.52068	5.9993	206.67	0.0459	10.0682	10.2881
	77->80	-0.15868					
	77->81	-0.37567					
	77->83	-0.10598					
10	75->79	0.41137	6.0849	203.76	0.1014	-24.6226	-24.4766
	76->79	-0.29442					
	77->79	0.13903					
	77->81	-0.37736					
	77->83	-0.10878					
	78->80	0.12748					
	78->86	0.13451					

[a]Number of the excited states; [b]Transitions; [c]Configuration-interaction coefficient; [d]Excitation energy; [e]Wavelength; [f]Oscillator strength; [g]Rotatory strength in velocity form (10^{-40} cgs); [h]Rotatory strength in length form (10^{-40} cgs).

Table S6. Key transitions, oscillator strengths, and rotatory strengths in the ECD spectrum of conformer **1b-5** at the B3LYP/6-31G (d, p) level of theory in MeOH with IEFPCM solvent model.

Num[a]	*Transition*[b]	*CI-coeff*[c]	*ΔE (eV)*[d]	*λ (nm)*[e]	*f*[f]	R_{vel}[g]	R_{len}[h]
1	77->80	-0.24284	4.7702	259.92	0.0576	-1.7703	-2.0849
	77->81	0.10884					
	78->79	0.64985					
2	78->80	0.20634	5.3255	232.81	0.0034	2.4707	2.6652
	78->81	0.46339					
	78->82	-0.45512					
	78->83	0.13127					
3	77->79	0.45823	5.4973	225.54	0.0167	-7.6761	-7.641
	78->80	0.49763					
	78->81	-0.17838					
4	78->80	0.13693	5.6308	220.19	0.0018	-3.0857	-3.1219
	78->81	0.41045					
	78->82	0.38508					
	78->83	-0.3288					
	78->84	0.14766					
	78->86	-0.16566					

5	78->81	0.19353	5.8078	213.48	0.0274	8.3341	8.4119
	78->82	0.3047					
	78->83	0.5273					
	78->84	0.12172					
	78->85	-0.18267					
	78->86	0.17857					
6	75->79	-0.18302	5.8891	210.53	0.1975	30.7437	30.8538
	77->79	0.30657					
	77->81	-0.19186					
	77->82	0.14953					
	78->80	-0.27292					
	78->84	0.10969					
	78->85	0.43368					
	78->87	0.12114					
7	77->79	0.10098	5.8934	210.38	0.0562	-10.6475	-11.0129
	77->80	-0.10604					
	78->80	-0.13725					
	78->82	-0.19585					
	78->83	-0.10760					
	78->84	0.52487					

	78->85	-0.32062					
8	75->79	0.17271	5.9516	208.32	0.0807	4.6029	4.8039
	77->79	-0.18143					
	77->80	0.16726					
	77->81	0.19406					
	77->82	-0.16532					
	78->80	0.13055					
	78->81	-0.11958					
	78->84	0.38546					
	78->85	0.34278					
	78->86	0.11188					
	78->87	0.11917					
9	75->79	0.40656	6.0538	204.80	0.0355	-13.9384	-13.5467
	76->79	-0.27309					
	77->80	-0.17423					
	77->81	-0.32535					
	77->82	0.28244					
10	76->79	0.59788	6.1067	203.03	0.0420	-7.1851	-7.6391
	77->79	-0.11166					

	77->81	-0.19253					
	77->82	0.15214					
	78->80	0.10183					
	78->86	0.17157					

[a]Number of the excited states; [b]Transitions; [c]Configuration-interaction coefficient; [d]Excitation energy; [e]Wavelength; [f]Oscillator strength; [g]Rotatory strength in velocity form (10^{-40} cgs); [h]Rotatory strength in length form (10^{-40} cgs).

Table S7. Key transitions, oscillator strengths, and rotatory strengths in the ECD spectrum of conformer **1b-6** at the B3LYP/6-31G (d, p) level of theory in MeOH with IEFPCM solvent model.

Num[a]	*Transition*[b]	*CI-coeff*[c]	*ΔE (eV)*[d]	*λ (nm)*[e]	*f*[f]	R_{vel}[g]	R_{len}[h]
1	77->80	-0.24295	4.7702	259.91	0.0576	-1.7694	-2.0844
	77->81	0.10870					
	78->79	0.64984					
2	78->80	0.20577	5.3254	232.81	0.0034	2.4745	2.6689
	78->81	0.46367					
	78->82	-0.45512					
	78->83	0.13117					
3	77->79	0.45807	5.4973	225.54	0.0167	-7.6894	-7.6543
	78->80	0.49797					
	78->81	-0.17794					
4	78->80	0.13671	5.6307	220.19	0.0018	-3.0853	-3.1217
	78->81	0.41051					
	78->82	0.38525					
	78->83	-0.32863					
	78->84	0.14768					
	78->85	-0.16563					

5	78->81	0.19335	5.8077	213.48	0.0274	8.334	8.412
	78->82	0.30453					
	78->83	0.52736					
	78->84	0.12175					
	78->85	-0.18289					
	78->86	0.17862					
6	75->79	-0.18258	5.8891	210.53	0.1967	30.7248	30.8348
	77->79	0.30615					
	77->81	-0.19173					
	77->82	0.14932					
	78->80	-0.27238					
	78->84	0.10928					
	78->85	0.43476					
	78->87	0.12129					
7	77->79	0.10166	5.8932	210.38	0.0566	-10.6489	-11.0144
	77->80	-0.10607					
	78->80	-0.13772					
	78->82	-0.19585					
	78->83	-0.10776					
	78->84	0.52507					

	78->85	-0.31975					
8	75->79	0.17284	5.9515	208.32	0.0810	4.6493	4.8506
	77->79	-0.18195					
	77->80	0.16716					
	77->81	0.19453					
	77->82	-0.16558					
	78->80	0.13095					
	78->81	-0.11957					
	78->84	0.38528					
	78->85	0.34219					
	78->86	0.11189					
	78->87	0.11899					
9	75->79	0.4066	6.0538	204.80	0.0357	-13.9674	-13.5765
	76->79	-0.27292					
	77->80	-0.17379					
	77->81	-0.32556					
	77->82	0.28247					
10	76->79	0.59777	6.1067	203.03	0.0420	-7.2048	-7.6598
	77->79	-0.11171					

	77->81	-0.19249					
	77->82	0.15204					
	78->80	0.10184					
	78->86	0.17213					

[a]Number of the excited states; [b]Transitions; [c]Configuration-interaction coefficient; [d]Excitation energy; [e]Wavelength; [f]Oscillator strength; [g]Rotatory strength in velocity form (10^{-40} cgs); [h]Rotatory strength in length form (10^{-40} cgs).

Table S8. Key transitions, oscillator strengths, and rotatory strengths in the ECD spectrum of conformer **1b-7** at the B3LYP/6-31G (d, p) of theory in MeOH with IEFPCM solvent model.

Num[a]	***Transition***[b]	***CI-coeff***[c]	***ΔE (eV)***[d]	***λ (nm)***[e]	***f***[f]	R_{vel}^{g}	R_{len}^{h}
1	77->80	0.25258	4.7691	259.97	0.0571	0.2394	-0.0898
	78->79	0.6498					
2	78->80	0.16054	5.3265	232.77	0.0035	1.5128	1.7114
	78->81	0.50577					
	78->82	0.42181					
	78->83	-0.14644					
3	77->79	-0.46115	5.4961	225.58	0.0168	-7.2807	-7.2646
	78->80	0.51036					
	78->81	-0.12755					
4	78->81	0.38874	5.6485	219.50	0.0022	-4.9383	-5.1015
	78->82	-0.36884					
	78->83	0.3772					
	78->84	-0.14707					
	78->86	0.15835					
5	78->81	-0.19462	5.7888	214.18	0.0173	7.5844	7.7416
	78->82	0.35212					
	78->83	0.48794					

	78->84	0.16960					
	78->85	0.18037					
	78->86	0.14656					
6	75->79	0.20457	5.8791	210.89	0.2547	22.6552	22.5799
	76->79	-0.17654					
	77->79	0.34460					
	77->81	0.20766					
	77->82	0.13702					
	78->80	0.31881					
	78->85	-0.33265					
7	78->81	0.10609	5.9046	209.98	0.0167	-7.2441	-7.4363
	78->82	-0.21850					
	78->84	0.62085					
	78->85	0.12111					
	78->87	-0.10205					
8	75->79	0.16025	5.9502	208.37	0.0330	-6.0802	-5.981
	76->79	-0.17080					
	77->79	0.12618					
	77->80	0.11929					
	77->81	0.14582					

	78->83	0.10757					
	78->84	0.17983					
	78->85	0.51121					
	78->86	-0.19402					
9	75->79	-0.24728	6.0174	206.04	0.0055	11.5704	12.2221
	76->79	0.4451					
	77->80	0.16902					
	77->81	0.35293					
	77->82	0.25969					
10	76->79	-0.27949	6.1219	202.52	0.0902	-31.0721	-32.0438
	77->79	-0.14191					
	77->81	0.22023					
	77->82	0.17031					
	78->80	-0.13965					
	78->83	-0.16782					
	78->86	0.47776					
	78->90	0.13765					

[a]Number of the excited states; [b]Transitions; [c]Configuration-interaction coefficient; [d]Excitation energy; [e]Wavelength; [f]Oscillator strength; [g]Rotatory strength in velocity form (10^{-40} cgs); [h]Rotatory strength in length form (10^{-40} cgs).

Table S9. Key transitions, oscillator strengths, and rotatory strengths in the ECD spectrum of conformer **1b-8** at the B3LYP/6-31G (d, p) level of theory in MeOH with IEFPCM solvent model.

Num[a]	***Transition***[b]	***CI-coeff***[c]	***ΔE (eV)***[d]	***λ (nm)***[e]	***f***[f]	R_{vel}[g]	R_{len}[h]
1	77->80	0.25259	4.7692	259.97	0.0571	0.2292	-0.1021
	78->79	0.64981					
2	78->80	0.16079	5.3265	232.77	0.0035	1.5066	1.7041
	78->81	0.50578					
	78->82	0.42172					
	78->83	-0.14638					
3	77->79	-0.46118	5.4962	225.58	0.0168	-7.2653	-7.2485
	78->80	0.51025					
	78->81	-0.12777					
4	78->81	0.38865	5.6487	219.49	0.0022	-4.951	-5.1144
	78->82	-0.36885					
	78->83	0.37731					
	78->84	-0.14699					
	78->86	0.15840					
5	78->81	-0.19471	5.7889	214.18	0.0172	7.5674	7.7237
	78->82	0.35225					
	78->83	0.48797					

	78->84	0.16942					
	78->85	0.18029					
	78->86	0.14648					
6	75->79	0.20490	5.8790	210.89	0.2549	22.6484	22.5707
	76->79	-0.17696					
	77->79	0.34478					
	77->81	0.20775					
	77->82	0.13708					
	78->80	0.31898					
	78->85	-0.33186					
7	78->81	0.62079	5.9048	209.97	0.0167	-7.2515	-7.4438
	78->82	-0.21836					
	78->84	0.62079					
	78->85	0.12143					
	78->87	0.10211					
8	75->79	0.16008	5.9502	208.37	0.0328	-6.0939	-5.993
	76->79	-0.17086					
	77->79	0.12569					
	77->80	0.11920					

	77->81	0.14526					
	78->83	-0.10747					
	78->84	-0.18022					
	78->85	0.51162					
	78->86	-0.19400					
9	75->79	-0.24709	6.0173	206.04	0.0056	11.6448	12.2976
	76->79	0.44528					
	77->80	0.16910					
	77->81	0.35288					
	77->82	0.25955					
10	76->79	-0.27929	6.1220	202.52	0.0903	-31.1506	-32.1251
	77->79	-0.14201					
	77->81	0.22073					
	77->82	0.17064					
	78->80	-0.13971					
	78->83	-0.16776					
	78->86	0.47745					
	78->90	0.13759					

[a]Number of the excited states; [b]Transitions; [c]Configuration-interaction coefficient; [d]Excitation energy; [e]Wavelength; [f]Oscillator strength; [g]Rotatory strength in velocity form (10^{-40} cgs); [h]Rotatory strength in length form (10^{-40} cgs).
